# Synthesis of super-biosafety hydrogen peroxide solution by ultrasonic cavitation

**DOI:** 10.1093/nsr/nwag218

**Published:** 2026-04-09

**Authors:** Qiao Wang, Feng Hong, Xuan Xia, Di Huang, Li Wang, Decheng Wang, Hongwei Huang, Xin Ying Kong, Yee Wen Teh, Minghui Lv, Tao Gao, Yingping Huang, Liqun Ye

**Affiliations:** College of Materials and Chemical Engineering, Key Laboratory of Inorganic Nonmetallic Crystalline and Energy Conversion Materials, China Three Gorges University, Yichang 443002, China; Engineering Research Center of Eco-environment in Three Gorges Reservoir Region, Ministry of Education, China Three Gorges University, Yichang 443002, China; Hubei Key Laboratory of Tumor Microenvironment and Immunotherapy, College of Basic Medical Sciences, China Three Gorges University, Yichang 443002, China; College of Materials and Chemical Engineering, Key Laboratory of Inorganic Nonmetallic Crystalline and Energy Conversion Materials, China Three Gorges University, Yichang 443002, China; Engineering Research Center of Eco-environment in Three Gorges Reservoir Region, Ministry of Education, China Three Gorges University, Yichang 443002, China; Engineering Research Center of Eco-environment in Three Gorges Reservoir Region, Ministry of Education, China Three Gorges University, Yichang 443002, China; Hubei Key Laboratory of Tumor Microenvironment and Immunotherapy, College of Basic Medical Sciences, China Three Gorges University, Yichang 443002, China; Beijing Key Laboratory of Materials Utilization of Nonmetallic Minerals and Solid Wastes, School of Materials Science and Technology, China University of Geosciences (Beijing), Beijing 100083, China; School of Chemistry, Chemical Engineering and Biotechnology, Nanyang Technological University, Singapore 637371, Singapore; School of Chemistry, Chemical Engineering and Biotechnology, Nanyang Technological University, Singapore 637371, Singapore; College of Materials and Chemical Engineering, Key Laboratory of Inorganic Nonmetallic Crystalline and Energy Conversion Materials, China Three Gorges University, Yichang 443002, China; College of Materials and Chemical Engineering, Key Laboratory of Inorganic Nonmetallic Crystalline and Energy Conversion Materials, China Three Gorges University, Yichang 443002, China; Engineering Research Center of Eco-environment in Three Gorges Reservoir Region, Ministry of Education, China Three Gorges University, Yichang 443002, China; College of Materials and Chemical Engineering, Key Laboratory of Inorganic Nonmetallic Crystalline and Energy Conversion Materials, China Three Gorges University, Yichang 443002, China; Engineering Research Center of Eco-environment in Three Gorges Reservoir Region, Ministry of Education, China Three Gorges University, Yichang 443002, China

**Keywords:** hydrogen peroxide, sonochemical synthesis, biosafety, argon, computational fluid dynamics

## Abstract

Hydrogen peroxide (H_2_O_2_) is the most widely used medical disinfectant product. However, the H_2_O_2_ solution synthesized by the industrial anthraquinone method currently contains trace amounts of heavy metal ions and toxic organic by-products, which pose a potential threat to human life and health. In this study, we propose the synthesis of super-biosafety H_2_O_2_ solution without any impurity through ultrasonic cavitation from pure water saturated with inert gases. The biosafety of H_2_O_2_ solution synthesized by ultrasonic cavitation (H_2_O_2_-U) was fully confirmed to be higher than that of commercial H_2_O_2_ (H_2_O_2_-C) by *in vitro* cell and *in vivo* mouse experiments. Computational fluid dynamic (CFD) simulation proved that the internal temperature of the argon (Ar) bubbles (710.50 K at 2.24 μs) is much higher than that of nitrogen (N_2_) bubbles (569.30 K at 2.26 μs). It indicated that Ar more favors water splitting to form H_2_O_2_, in good agreement with the experimental results. Both radical experiment and hydrodynamic simulations disclose the reaction essence that sonication-induced local high temperature and pressure split pure water to ·OH and ·H radicals, which subsequently couple to generate H_2_O_2_ and H_2_. This work proposes an infusive methodology for the efficient synthesis of super biosafety H_2_O_2_ solution, and presents the first real-life applications.

## Teaser

Ultrasonic cavitation was found to efficiently synthesize H_2_O_2_ solution under inert gases without the addition of metal ions and organic compounds, only by the water decomposition and ·OH radical combination, thus displayed super *in vitro* and *in vivo* biosafety.

## INTRODUCTION

Hydrogen peroxide (H_2_O_2_), recognized as a strong oxidizing agent, plays a pivotal role in water treatment, environmental disinfection, and biomedical applications ranging from pharmaceutical synthesis to clinical therapeutics [1]. It exhibits antibacterial potential in surface disinfection, sterilization, and antiviral applications (such as suppression of influenza virus and COVID-19 virus) [2], which has widespread use in hospitals and other medical facilities as a cleaning agent. In therapeutics, H_2_O_2_ targets the tumor microenvironment through chemodynamic therapy, catalyzing the generation of hydroxyl radicals to induce apoptosis of cancer cells. Given the extensive use of H_2_O_2_, it inevitably enters the human body through inhalation, injection, and skin contact, while the impurities in H_2_O_2_ pose significant health risks to the human body, as residual metal ions or organic impurities may catalyze the generation of toxic free radicals or induce nonspecific damage. Therefore, ensuring extremely high biological safety of H_2_O_2_ through stringent purity standards is one of the most critical indicators in biomedical applications, aimed at mitigating potential health risks to the human body, which underscores the critical importance of high-purity preparation and impurity control.

Presently, H_2_O_2_ is mainly synthesized via the anthraquinone oxidation process in industry [3,4]. This method entails substantial energy consumption, and has potential safety hazards (toxic by-products such as 2-ethylanthraquinone, phosphate, and aromatic hydrocarbons, inorganic ions such as Cd, Pd, As, and Pb), thus not suitable for production of H_2_O_2_ used as a medical disinfectant. Recent research has explored safer preparation methods, namely electrochemical and photochemical approaches [5–7]. By the electrochemical methods, a common research scheme is to use 2e^−^ oxygen reduction (ORR) or 2e^−^ water oxidation (WOR) to prepare H_2_O_2_ [6,8–12]. If H_2_ and O_2_ are delivered to the anode and cathode of a porous solid electrolyte separation, respectively, then the H_2_O_2_ yield can reach 3.4 mmol cm^−2^ h^−1^ from the recombination of electrochemically generated H^+^ and HO_2_^−^ [13]. In photochemistry, structural modifications by introducing both oxidation and reduction sites have enabled efficient H_2_O_2_ production in both WOR and ORR dual channels [14–17]. Electrochemical and photochemical methods offer portable synthesis solutions for H_2_O_2_, with enhanced production efficiency [18,19]. However, the low purity of H_2_O_2_ poses a significant drawback for current photochemical and electrochemical methods, as they necessitate catalysts and sometimes organic reagents. The addition of chemical substances (such as phosphate, potassium hydroxide, and organic sacrificial agents) or potential leaching from catalysts (such as heavy or transition metal ions Fe, Cu, Co, Pd, Pt, Rh, Ru, and Ir) [20,21], profoundly compromises the purity of H_2_O_2_, and pose significant health risks to the human body, which limits the application of H_2_O_2_, especially in the medical field. Therefore, it is very necessary to develop an effective methodology that can generate high-purity and super-biosafety H_2_O_2_ directly from pure water without adding any other chemical substances and solvent.

To achieve the above goal, using water as the single reactant may be prospective, while the additional energy introduction methods need to be considered. Recently, water in microdroplets were found to spontaneously generate H_2_O_2_ without added catalysts, which was attributed to an intense electric field (∼10^9^ V/m) at the air–water interface, spontaneously splitting water molecules to generate ·OH radicals and subsequently forming H_2_O_2_ [22,23]. By contact electrocatalytic (CEC) synthesis, H_2_O_2_ could be spontaneously produced via the recombination of ·OH radicals generated from hydroxyl groups on the solid surface upon contact with water [24,25]. The yield, concentration, and equipment operation stability of these methods still need to be improved. As a commonly used energy source, ultrasound was first discovered to have chemical effects by Richard and Loomis in 1927 [26]. Since then, enormous research has been conducted on sonochemistry [27]. Ultrasonic cavitation, involving higher ultrasonic energy in the liquid and internal cavitation bubbles, facilitates localized extreme conditions such as high temperature, high pressure, as well as micro-jet [28]. These features provide favorable conditions for chemical catalysis and material dispersion, and thus are widely used in biology, medicine, health, and instrument cleaning [29,30]. For the production of H_2_O_2_, researchers have explored the effects of frequencies and atmospheres on the productivity of H_2_O_2_ by ultrasonic cavitation [28,31–33], but the yield (only 10 μmol L^−1^ h^−1^) was relatively low at the current optimal conditions [34]. To better utilize the advantage of ultrasound cavitation in H_2_O_2_ generation without the addition of metal ions and organic compounds, it is anticipated to thoroughly uncover the reaction mechanism by experiments combined with hydrodynamic simulations to achieve efficient H_2_O_2_ output under cavitation, which is hopeful to be applied directly to the medicine and environment with super biosafety.

Here, we reported a method to efficiently produce super-biosafety H_2_O_2_ solution without any impurity by ultrasonic cavitation with inert gases. The purity of H_2_O_2_ in this study was proved comparable to AR grade. The optimal synthesis conditions for H_2_O_2_ production were investigated by experiments and computational fluid dynamic (CFD) simulation. More importantly, H_2_O_2_ solution synthesized by ultrasonic cavitation (H_2_O_2_-U) was confirmed to have high biosafety by *in vitro* and *in vivo* experiments, compared with the commercial H_2_O_2_ (H_2_O_2_-C). The transfer of H and O atoms during the decomposition of water is also systematically studied through isotopic labeling experiments. Experiments and CFD simulations proved that the induced local high temperature and pressure by sonication is essential for pure water to split into ·OH and ·H radicals, which subsequently couple to generate H_2_O_2_ and H_2_. This work provides an efficient methodology for the synthesis of super-biosafety H_2_O_2_ solution without the addition of metals or organic reagents, and demonstrates the sonochemical H_2_O_2_ generation mechanism by experiments and simulation, providing a theoretical foundation and expanded perspective for subsequent biological applications and research in ultrasonic chemistry.

## RESULTS AND DISCUSSION

### H_2_O_2_ synthesis by ultrasonic cavitation

The synthesis of H_2_O_2_ by ultrasonic cavitation, electrocatalysis, and semiconductor photocatalysis was investigated and compared (Fig. S1). To obtain H_2_O_2_ solution with high purity, the catalyst and organic substances should not be involved in the synthesis process, which can be realized by the sonochemical route. In the process of ultrasonic cavitation, the cavitation bubble has a cycle of contraction and expansion with the fluctuations of ultrasonic pressure. Ultimately, the cavitation bubble collapses when the critical pressure is reached. This will promote the production of the chemical ·OH (Fig. S1c), facilitating the successful preparation of H_2_O_2_. To confirm the synthesis of AR-grade H_2_O_2_ by sonochemistry, we collected H_2_O_2_ solutions with the same concentration but different preparation methods for Rhodamine B (RhB) degradation. The findings showed that H_2_O_2_ synthesized by sonochemistry was capable of completely degrading RhB, while the degradation effect through the traditional preparation method was not obvious (Fig. S1e). As the outcomes of sonochemical processes can be significantly altered by changing the reaction conditions, several important parameters, including atmospheric conditions, ultrasonic power, solution pH, and solution temperature, were investigated in this study to ascertain the impacts of the external environment on the production efficiency of H_2_O_2_.

**Figure 1.**
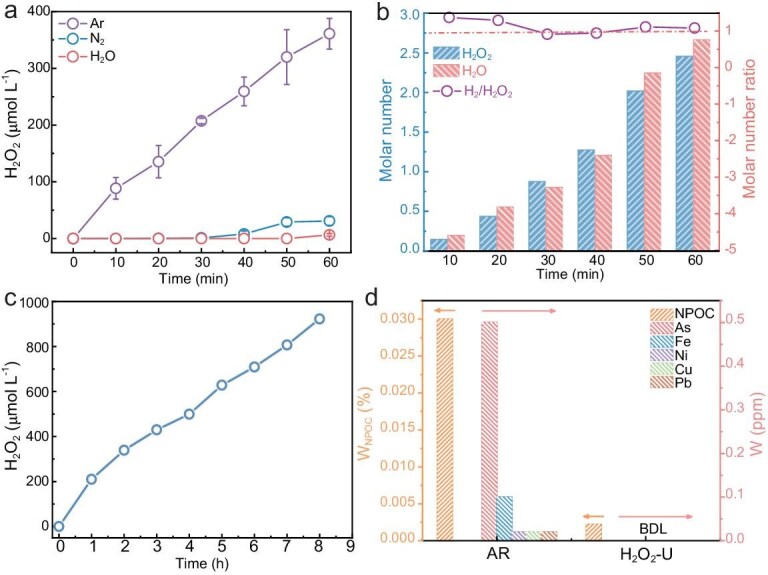
H_2_O_2_ yield by ultrasonic cavitation. (a) H_2_O_2_ production under the Ar, N_2_, and H_2_O vapor atmospheric conditions (10 mL H_2_O, *f* = 40 kHz, *P* = 2.77 W cm^−2^). (b) The ratio of moles of H_2_ to H_2_O_2_. (c) The generation of H_2_O_2_ after sonication for 8 h of the H_2_O_2_ solutions (Ar, 10 mL H_2_O, *T* = 20°C, pH = 7.0, *f* = 40 kHz, *P* = 2.77 W cm^−2^). (d) Mass fractions of NPOC and metal ions (As, Fe, Ni, Cu, and Pb) in the H_2_O_2_ with purity of international standard analytical reagent levels (AR) and ultrasonic cavitation synthesized H_2_O_2_ (H_2_O_2_-U). BDL stands for below detection limit.

The sonochemical synthesis experiment was conducted in a sealed bottle (Fig. S1d), which allowed easy regulation of the specific atmosphere and sonication conditions. The concentration of H_2_O_2_ can be obtained by the standard curve method (Fig. S2). The effects of dissolved gases during the ultrasonic synthesis of H_2_O_2_ under the Ar, N_2_, and H_2_O vapor atmospheric conditions were examined. After 60 min of ultrasonic synthesis, the concentration of H_2_O_2_ could reach ∼360 μmol L^−1^ under the Ar condition, and the corresponding energy consumption per unit was 3.27 kWh g^−1^. The H_2_O_2_ yields under N_2_ and water vapor conditions could only reach about 35 μmol L^−1^ h^−1^ and 9 μmol L^−1^ h^−1^ within 60 min, respectively (Fig. 1a). The moles of H_2_O_2_ and H_2_ were measured at varying intervals (Fig. 1b), revealing that the moles of H_2_O_2_ and H_2_ grew with time, and the ratio of the moles of H_2_ to H_2_O_2_ was close to 1, aligning with the theoretical outcomes 2H_2_O → H_2_O_2_ + H_2_. The results showed that the highest efficiency of H_2_O_2_ production was achieved under Ar conditions. Notably, by prolonging the acoustic irradiation period to 8 h, the concentration of H_2_O_2_ accumulates up to 992 μmol L^−1^ (Fig. 1c). We extended the dissolved gas to He, Ne, and Kr during the ultrasonic synthesis of H_2_O_2_ (Fig. S3), and the Ar and Kr atmospheres displayed the highest production concentration of H_2_O_2_, compared to other noble gases. Because the Ar gas is more economical than Kr gas, we further mainly synthesized H_2_O_2_ under Ar condition. Under Ar atmosphere, when the output ultrasonic power increased from 0.77 to 2.77 W cm^−2^, the productivity of H_2_O_2_ increased by 8 times within 60 min (Fig. S4). This can likely be attributed to the fact that with the increase in ultrasonic power, the number of cavitation bubbles (or hotspots) and the temperature inside a single cavitation bubble gradually increased. The sonochemical total decomposition of water results in the generation of H_2_O_2_ and H_2_. The amount of H_2_ produced was determined to be 5.28 and 0.35 μmol after 2 h under Ar and N_2_ conditions, respectively (Fig. S5a).

The effect of different water bath temperatures on the ultrasonic generation of H_2_O_2_ was then explored (Fig. S6a). Results indicated a rise and subsequent decline in H_2_O_2_ yield correlating with increasing temperature, with the optimal H_2_O_2_ yield of 351 μmol L^−1^ h^−1^ achieved at 20°C. Contrary to previous studies suggesting a negligible impact of temperature adjustments on H_2_O_2_ generation efficiency [34–36], our experimental findings reveal a distinct correlation. Specifically, when the temperature is lower than 20°C, the H_2_O_2_ yield increases with the increase in temperature. This might be attributed to the fact that rising temperature engenders a gradual increase in the number and collapse rate of cavitation bubbles during the ultrasonic process, thus enhancing the production efficiency of ·OH, and the effective combination of ·OH to generate H_2_O_2_. Conversely, when the temperature is >20°C, the production rate of H_2_O_2_ decreases with the increase of temperature, which should be attributed to the occurrence of a negative effect. Drawing on Laxim’s viewpoint [37], with the gradual increase of water temperature, the content of water vapor in the confined system also gradually increases, leading to the infusion of water vapor into developing cavitation bubbles. This incorporation lessens the impact of collapsing cavitation bubbles, diminishing the ultrasonication efficiency, and consequently, reducing H_2_O_2_ production.

Solution pH is one of the pivotal factors that affect the efficiency of the experiment. This study investigated the effect of pH on the generation of H_2_O_2_ by adjusting the initial pH of the solution (Fig. S6b). Earlier studies have shown that the initial pH of the solution (3, 7, 11) does not affect the efficiency of H_2_O_2_ generation [38,39]. However, as indicated by Fig. S6b, the H_2_O_2_ generation rate remains almost constant with a pH range of 4–10. At pH 2, the production rate of H_2_O_2_ was considerably lower. This reduction is attributed to the hindered recombination of ·OH under acidic conditions, thereby suppressing the prevalent formation of H_2_O_2_. Similarly, the production efficiency of H_2_O_2_ was also relatively low under the strong alkaline environment of pH 12. This is because under alkaline conditions, H_2_O_2_ deprotonates to generate HO_2_^−^ (H_2_O_2_/HO_2_^−^ p*K*a = 11.75 Eq. 1). According to the reaction, H_2_O_2_ reacts with HO_2_^−^ to generate H_2_O and O_2_ (Eq. 2). Under alkaline conditions, the rate of self-decomposition of H_2_O_2_ increases, culminating in a decrease in H_2_O_2_ production [40]. In addition, the decomposition of H_2_O_2_ can be enhanced under extremely acidic or alkaline condition, thus influencing the net yield during the H_2_O_2_ generation process (Fig. S7).


(1) \begin{eqnarray*}
{\mathrm{H}}{{\mathrm{O}}}_2^ - + {{\mathrm{H}}}_2{{\mathrm{O}}}_2 \to {{\mathrm{H}}}_2{\mathrm{O}} + {{\mathrm{O}}}_2 + {\mathrm{O}}{{\mathrm{H}}}^ - ,
\end{eqnarray*}

(2) \begin{eqnarray*}
2{{\mathrm{H}}}_2{{\mathrm{O}}}_2 \to 2{{\mathrm{H}}}_2{\mathrm{O}} + {{\mathrm{O}}}_2.
\end{eqnarray*}

Subsequently, a comparison of different water qualities was conducted (Fig. S8), revealing that H_2_O_2_ can be generated under tap water, plain water, and ultrapure water with only a slight difference in the production rate. The highest yield of H_2_O_2_ was obtained in ultrapure water as it contains the fewest impurities, thus exerting the smallest impact on the acoustic production of H_2_O_2_. This means that the sonochemical decomposition of water for H_2_O_2_ production is suitable for extensive water source.

Purity is the most important factor for the application performance of H_2_O_2_ in practice besides concentration, and the addition of any chemical reagent can significantly compromise the purity of H_2_O_2_, as involved in electrochemical and photochemical techniques. To compare the purity of H_2_O_2_ synthesized by different chemical processes in this study, we examined the organic carbon content and metal ion content in the H_2_O_2_ solution synthesized by acoustic chemistry, photochemical, and electrochemical methods by non-purgeable organic carbon (NPOC) method and inductively coupled plasma technique, controlling the concentration at around 1 mmol L^−1^ [41,42]. As shown in Fig. 1d, higher concentrations of NPOC and metal ions (especially As and Fe) were detected in the H_2_O_2_-C. In contrast, no metal ions were detected and only a low mass fraction of organic carbon was present in ultrasonic cavitation synthesized H_2_O_2_ (H_2_O_2_-U) solutions, the purity of which was up to AR grade without any need for further purification. In addition, in Fig. S9, photochemical and electrochemical routes, due to the addition of chemical reagents, yielded H_2_O_2_ solutions with notable metal ions and organic carbon mass fractions of 0.81% and 2.73%, which must be subsequently purified to meet AR grade according to international standards (specific values are detailed in the Table S1). The above results imply that the ultrasonic cavitation-produced H_2_O_2_ has significantly high purity, and the absence of metal ions with extremely low proportion of organic by-products renders the produced H_2_O_2_ potentially high biosafety, and thus it can be used for environmental purification and biomedical applications in practice.

### Biosafety of ultrasonically synthesized H_2_O_2_ by *in vitro* experiments

**Figure 2.**
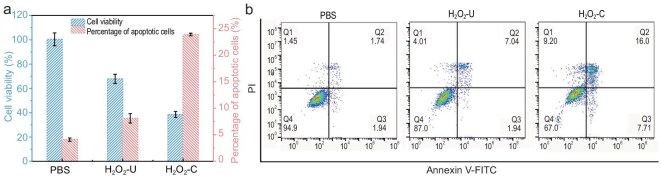
The biosafety measurements of H_2_O_2_ prepared by sonication (H_2_O_2_-U), and commercial H_2_O_2_ (H_2_O_2_-C) by *in vitro* cell experiments. (a) The cell viability of A549, and the percentage of apoptosis in A549 cells treated by H_2_O_2_ prepared by ultrasonic cavitation (H_2_O_2_-U), compared with commercial H_2_O_2_ (H_2_O_2_-C) at a concentration of 250 μM for 24 h, with PBS as the control. (b) The apoptosis in A549 cells treated by H_2_O_2_ prepared using different methods. The data were assessed using Annexin V-FITC/PI dual staining. Q1: Annexin V⁻/PI⁺, necrotic or mechanically damaged cells; Q2: Annexin V⁺/PI⁺, late apoptotic/necrotic cells; Q3: Annexin V⁺/PI⁻, early apoptotic cells; Q4: Annexin V⁻/PI⁻, viable cells.

To confirm the biosafety of H_2_O_2_, we investigated the viability and apoptosis of cells treated by ultrasonically synthesized H_2_O_2_ (H_2_O_2_-U) with other methods (photocatalysis, electrocatalysis), as well as the commercial H_2_O_2_ (H_2_O_2_-C). As shown in Fig. 2a and Fig. S10, after treating A549 cells with H_2_O_2_ prepared by five different methods for 24 h, cell viability was significantly reduced in all cases. Moreover, the H_2_O_2_ prepared by the CdS exhibited the strongest cytotoxicity, reducing cell viability to below 10%; the H_2_O_2_-C was the next most toxic, reducing cell viability to around 40%; the H_2_O_2_ prepared by photocatalysis (H_2_O_2_-P), electrocatalysis (H_2_O_2_-E), and sonication (H_2_O_2_-U) showed similar cytotoxic effects, reducing cell viability to around 70%. When the treatment time was extended to 29 h (Fig. S11), cell viability further decreased, but the relative cytotoxicity strength of the H_2_O_2_ prepared by the five methods remained unchanged, ranking from strongest to weakest as CdS, H_2_O_2_-C, H_2_O_2_-P, H_2_O_2_-E, and H_2_O_2_-U. The morphology of A549 cells treated with H_2_O_2_ prepared by different methods for different durations is shown in Fig. S11. The results indicate that H_2_O_2_ synthesized by sonication possesses higher biosafety than other methods, which is consistent with the purity results in Fig. 1d and Fig. S9.

The cell apoptosis process was also studied by treating the A549 cells using H_2_O_2_-U, H_2_O_2_-C, H_2_O_2_-P (with non-metallic catalyst), H_2_O_2_-E, and CdS (H_2_O_2_-P with metal catalyst), respectively. After treating A549 cells with H_2_O_2_ prepared by five different methods for 24 h, the apoptosis of the cells was detected. In Fig. 2a, b and Fig. S10, the results showed that the total proportion of early and late apoptotic cells in each treatment group was significantly higher compared to the PBS (phosphate buffered saline) group. The methods causing apoptosis in descending order of proportion were CdS, H_2_O_2_-C, H_2_O_2_-E, H_2_O_2_-P, and H_2_O_2_-U. The results further confirmed that the ultrasonically synthesized H_2_O_2_ from pure water saturated with inert gases without any impurity has super biosafety, rendering it enormous potential for direct application to the medicine and environment.

### H_2_O_2_-U induced less injury on the stomach mucosa of mice than H_2_O_2_-C

**Figure 3.**
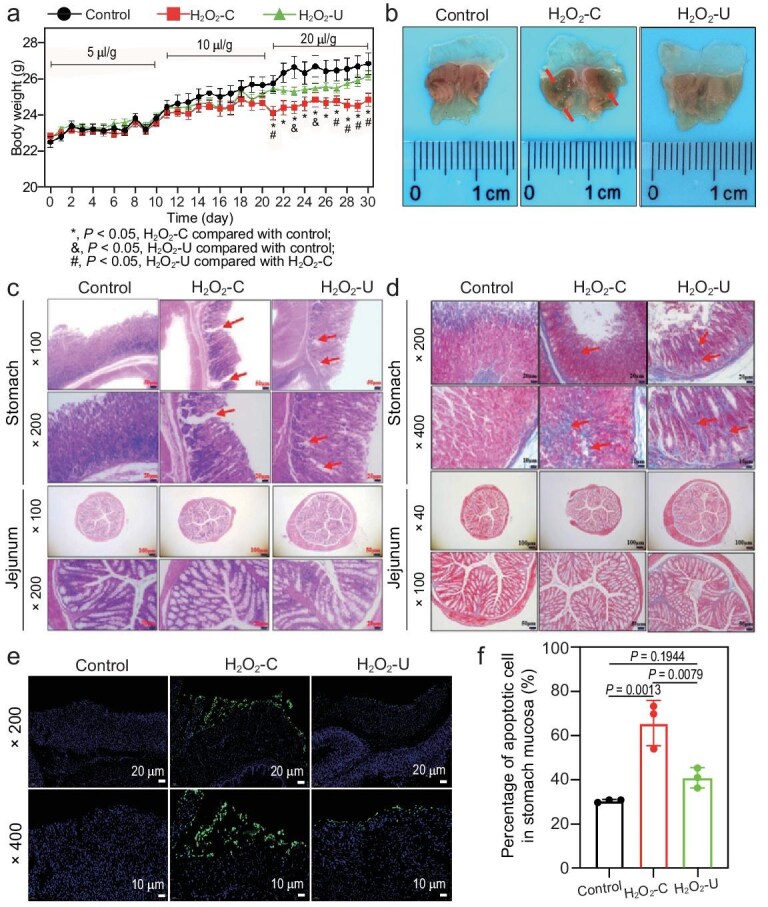
H_2_O_2_-U induced less injury on the stomach mucosa of mice than H_2_O_2_-C. (a) Ultra-pure H_2_O_2_ (H_2_O_2_-U) showed less weight loss than commercial H_2_O_2_ (H_2_O_2_-C). The C57BL/6 J mice in three groups were orally administrated (500 μmol L^−1^, once daily by gavage, 5 μL g^−1^ (body weight) per day for the first 10 days, up-titrated to double dosage every 10 days) for consecutive 30 days (*n* = 10 per group). (b) Gross observation of dissected stomach after mice sacrificing at the end of experiments. (c) Hematoxylin eosin staining of stomach and jejunum mucosa in H_2_O_2_-U, H_2_O_2_-C, and control groups (*n* = 3 per group). (d) Masson’s trichrome stain of stomach and jejunum mucosa in H_2_O_2_-U, H_2_O_2_-C, and control groups (*n* = 3 per group). (e) TUNEL stain of stomach mucosa in H_2_O_2_-U, H_2_O_2_-C, and control groups (*n* = 3 per group). (f) Statistical analysis of apoptotic cells in three groups (the green fluorescence-stained cells were regarded as the apoptotic cells). The percentage of green (apoptotic cell)/blue (DAPI-stained nucleus of normal cells) in three view fields under the microscope will be calculated and statistically analyzed. *, *P* < 0.05, H_2_O_2_-C compared with control; &, *P* < 0.05, H_2_O_2_-U compared with control; #, *P* < 0.05, H_2_O_2_-U compared with H_2_O_2_-C.

Additionally, to better compare the biosafety of synthesized H_2_O_2_ by sonochemistry (H_2_O_2_-U) with commercial H_2_O_2_ (H_2_O_2_-C), we conducted toxicity tests on mice *in vivo*. H_2_O_2_ is clinically used not only for skin wound washing but also for mucosal and oral rinsing, where unintended ingestion cannot be completely avoided [43]. The remnant of H_2_O_2_ in gastric tract brings more concern on biosafety. Hence, we used an animal model with oral gavage to evaluate its long-term toxicity. In our animal experiment for 30 days, there was no significant difference in body weight among the groups. With dosage up-titration of both H_2_O_2_-C and H_2_O_2_-U, the slowing down of weight gain was observed in H_2_O_2_-C and H_2_O_2_-U groups. The body weight was much greater in H_2_O_2_-U than in H_2_O_2_-C (Fig. 3a). After evisceration, there was no macroscopic difference in the liver, jejunum, and colon among the three groups (Fig. S12a). There was no statistical difference in organ weights of the liver, kidney, and heart, while serum markers of liver (ALT, AST) and renal function (BUN, sCr) were shown to be not altered among the groups (Fig. S12b–e). Strikingly, the gross morphological profile of the stomach was obviously different among the groups. There were distinct stomach ulcers, thin and atrophic mucosa, and fewer gastric folds in the H_2_O_2_-C group compared with the vehicle group, while there was less injury in the H_2_O_2_-U group than in the H_2_O_2_-C group (Fig. 3b).

In the H&E stain of the stomach, there were consistent results under microscopical observation among the groups (Fig. 3c). Compared with the vehicle group, there was thinning, shortenning, loss of mucosa in the H_2_O_2_-C group which was more severe than in the H_2_O_2_-U group. Intriguingly, there was no differential alteration of mucosa of jejunum among the groups (Fig. 3c). In Masson’s trichrome stain of the stomach, there was significantly increased blue regions in stomach interstitium, suggesting that fibrosis occurred both in H_2_O_2_-C and H_2_O_2_-U groups, with no obvious difference between them (Fig. 3d). Meanwhile, in H&E stain of colon, there was thinning, shortening, loss of mucosa in both H_2_O_2_-C group and H_2_O_2_-U group relative to the control group. In Masson’s trichrome stain of the colon, there is no obvious fibrosis for the three groups (Fig. S13).

To explore the underlying mechanism of the H_2_O_2_-induced injury on gastric mucosa, we used TUNEL stain (green fluorescence methods) to validate the apoptosis occurrence in three groups (Fig. 3e). The area of green fluorescence indicated the apoptotic cells of mucosa in the stomach. There was a significantly increased apoptosis percentile of mucosal cells in H_2_O_2_-C than in H_2_O_2_-U and in the control group (Fig. 3f). There is slightly increased apoptosis in H_2_O_2_-U which had no statistically difference with the control group. In total, H_2_O_2_-C induced prominent cell death of stomach mucosa through an apoptosis style. H_2_O_2_-U had a much less toxic effect on stomach mucosa than H_2_O_2_-C. Because the catalysts used for photocatalytic synthesis and electrocatalytic synthesis of H_2_O_2_ are mainly organic materials, and the release of organic impurities from catalysts to the H_2_O_2_ solution may have great impact for the medical use, we further investigated the influence of ethylene diamine tetraacetic acid (EDTA) (the common organic stabilizer used in H_2_O_2_ solution) exposure *in vitro* (A549 cell). In Fig. S14, prolonged EDTA exposure *in vitro* could bring cellular injury, decrease cell viability, and induce mild apoptosis, while short-term EDTA exposure bring little influence to cell viability. The results further emphasize the importance of high-purity H_2_O_2_ solutions without organic impurities. Without organic stabilizers, the H_2_O_2_-U still demonstrated excellent stability (Fig. S15).

### CFD simulations of ultrasonic cavitation process

There are more influencing factors affecting the production efficiency of H_2_O_2_, including the specific heat capacity ratio of the gas, the solubility, the temperature at the time of bubble bursting, and the pressure [31]. The *γ* values of He, Ne, Ar, and Kr are 1.63, 1.66, 1.66, and 1.66, respectively, and the *γ* values of several noble gases are relatively close to each other. To further substantiate and analyze the rationale behind the above experimental results, CFD simulations were carried out to compare the temperature, pressure, and volume fraction inside the bubbles under ultrasonic conditions. The CFD simulation involves an ultrasonic bubble filled up with Ar, N_2_, and water vapor within an infinite liquid domain, respectively, where an ultrasonic frequency of 40 kHz and pressure amplitude of 4×10^6^ Pa (4.0 p_α_) are exerted on the bottom of the reactor, according to the experimental condition. The simulation setup, model validation and mathematical model are delineated in Supporting Information (SI) Section 2, Section 3, and Figs S16 and S17.

**Figure 4.**
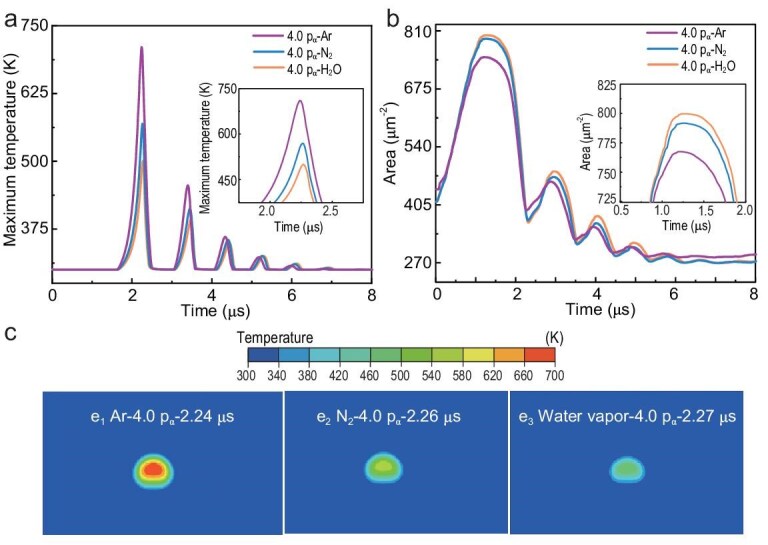
Physical effects of different collapsing bubbles obtained by CFD computation (ultrasound frequency *f* = 40 kHz, bubble radius *R* = 10 μm, pressure magnitude *P* = 4.0 p_α_). (a) Local maximum temperature with computing time. (b) Bubble area variation with computing time. (c) Peak temperature distribution for different gases.

The effect of pressure during the ultrasonic cavitation was firstly investigated (Fig. S18). As observed, the maximum temperature is elevated with the increase of the pressure amplitude, and the same trend is also found for the pressure variation near the hotspot where the bubble collapses. Under Ar atmosphere, the red color area calculated using the pressure amplitude of 4.0 p_α_ is much larger than that of 1.0 p_α_ (Fig. 4 and Fig. S18), implying that a larger hotspot and more cavitation bubbles are expected in the former case. The bubble kinetic process under 1.0 and 4.0 p_α_ were also simulated in Fig. S19. The higher pressure promotes the bubble reaching the largest size earlier, after which the bubble shrinks to the maximum extent, and the maximum temperature is recorded at the same time. In Fig. 4a and c, the simulation results (compared using the pressure amplitude of 4.0 p_α_) indicate that the bubble filled up with N_2_ reached its maximum temperature of 569.30 K at 2.26 μs, while water vapor reached the maximum temperature of 500.10 K at 2.27 μs. Under Ar condition, the bubble reached its maximum temperature of 704.35 K at 2.23 μs, and the size of the Ar bubbles is much smaller than that of N_2_ and water vapor (Fig. 4b). This suggests that the Ar bubble experiences more intense contraction and expansion processes than N_2_ and water vapor bubbles, which is in good accordance with the results as shown in Fig. 1a and Fig. S3, where a much higher temperature is generated for the Ar atmosphere. Relative to the temperature of bulk solution, a higher peak bubble temperature generates a larger instantaneous flux of H_2_O_2_ per collapse event, as corroborated in Fig. S6a. However, when the bulk temperature exceeds 20°C, H_2_O_2_ undergoes intensified thermal decomposition in the liquid phase. Simultaneously, the content of water vapor in the confined system also gradually increases, leading to the infusion of water vapor into developing cavitation bubbles. Photographs of acoustic chemiluminescence in water under different atmosphere conditions were acquired, enabling the visualization and comparison of the cavitation field’s intensity magnitude. As illustrated in Fig. S20, the intensity of acoustic chemiluminescence in Ar is stronger than that in N_2_ conditions, which proves that the intensity of the cavitation field is superior in Ar conditions [44].

We further compared the CFD simulation results involving an ultrasonic bubble filled with He, Ne, Ar, and Kr within an infinite liquid domain, respectively. Upon collapse, the bubble filled with Ar or Kr had a higher temperature (704.35 or 706.10 K) than those filled with He or Ne (567.50 or 672.19 K) (Fig. S21). In addition, the local pressure of Ar is close to that of Kr, reaching over 7.0 MPa, higher than those of He and Ne (Fig. S22). Such conditions are advantageous for yielding H_2_O_2_ through sonication, which is consistent with the trend of H_2_O_2_ generation ability in Fig. 1a. Combined with the predicted hydrodynamic characteristics and experimentally measured results under different gases, the extreme local temperature and pressure are decisive factors for the H_2_O_2_ production in this issue. When multi-bubble systems are considered, the same trend is observed in the CFD results (Fig. S23 and Table S2).

### Mechanistic investigation

**Figure 5.**
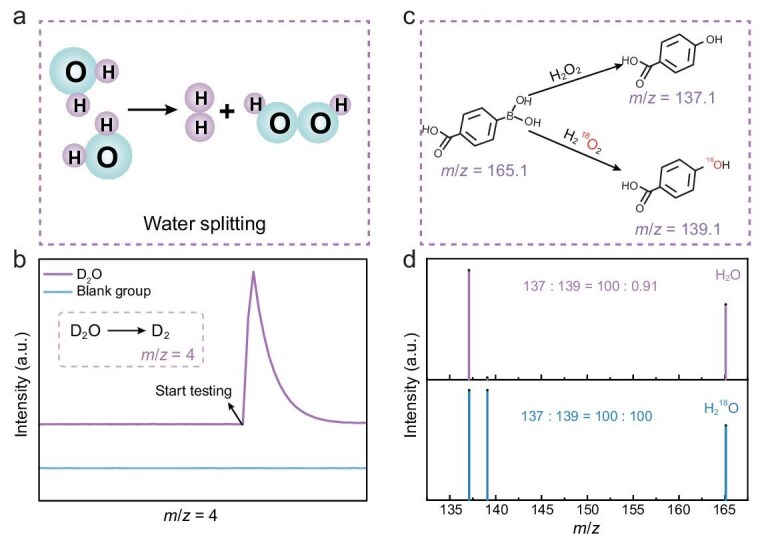
Verification of the source of H_2_O_2_ and H_2_. (a) Schematic diagram of total decomposition of water. (b) Mass spectra of D_2_. (c) Scheme for H_2_O_2_/H_2_^18^O promoted deacylation of 4-carboxyphenylboronic acid. (d) Mass spectra of 4-hydroxybenzoic acid after H_2_^18^O treatment.

Numerous studies have shown that the sonolysis of water to produce H_2_O_2_ is an extremely complex process. While several related chemical reactions have been proposed by researchers, the predominant chemical process consists of only two steps: initially, the ultrasonic decomposition of water produces ·OH and ·H (Eq. 3) radicals [45], and then two ·OH are combined into H_2_O_2_ (Fig. 5a). However, the ·OH generated during cavitation rapidly interacts with ·H to form H_2_O, and only around 20% of the radicals can be coupled according to the reaction equation (Eq. 4) [46]. This scarcity in radical coupling explains the diminished efficiency in the acoustic chemical generation of H_2_O_2_.


(3) \begin{eqnarray*}
{{\mathrm{H}}}_2{\mathrm{O}} + ))) \to \cdot {\mathrm{H}} + \cdot {\mathrm{OH,}}
\end{eqnarray*}

(4) \begin{eqnarray*}
\cdot {\mathrm{OH}} + \cdot {\mathrm{OH}} \to {{\mathrm{H}}}_2{{\mathrm{O}}}_2,
\end{eqnarray*}

(5) \begin{eqnarray*}
\cdot {\mathrm{H}} + \cdot {\mathrm{H}} \to {{\mathrm{H}}}_2,
\end{eqnarray*}

(6) \begin{eqnarray*}
\cdot {\mathrm{OH}} + \cdot {\mathrm{H}} \to {{\mathrm{H}}}_2{\mathrm{O}}{\mathrm{.}}
\end{eqnarray*}

The water splitting results in the production of stoichiometric H_2_ and H_2_O_2_, demonstrating that the O atoms in H_2_O_2_ and the H atoms in H_2_ originate from H_2_O [47]. For further confirmation, isotopic labeling experiments were conducted. When H_2_O is replaced with D_2_O and subjected to sonication, a remarkable peak attributing to D_2_ (*m/z* = 4) is detected by process monitoring quadrupole mass spectrometer (PM-QMS), while the blank group showed no fluctuation at *m/z* = 4 (Fig. 5b). It corroborates that the H atoms in H_2_ are indeed derived from the H_2_O. To ascertain the elemental origin of the as-generated H_2_O_2_, an O isotope tracing experiment was designed to further explore the sources of O in the generated H_2_O_2_. H_2_O_2_ oxidizes 4-carboxyphenylboronic acid to 4-hydroxybenzoic acid (Fig. 5c), and the different products can be observed based on the mass-to-charge ratio [48,49]. The experimental procedure was performed with 4 mL of H_2_^18^O and compared with 4 mL of H_2_O as a solution. If the O in H_2_O_2_ is derived from H_2_O, then ^18^O can be detected in the corresponding 4-carboxyphenol. After isotopic treatment (Fig. 5d), the solution without the addition of H_2_^18^O was normalized to 4-carboxyphenylboronic acid *m/z* = 137.1, displaying a relative abundance of 0.9% at *m/z* = 139.1. Conversely, in the solution with the addition of 4 mL of H_2_^18^O, the intensity of the mass spectral peaks at 139.1 *m/z* was enhanced, with a relative abundance of 100% (Fig. S24), which indicated that the O atoms of H_2_O_2_ are indeed derived from H_2_O.

**Figure 6.**
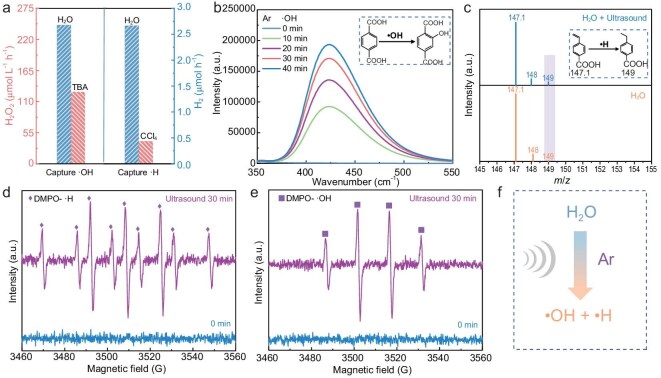
Detection of active species. (a) Effect of capturing ·OH and ·H on H_2_O_2_ and H_2_ production rates (5 mmol CCl_4_ capture ·H, 0.1 mol L^−1^  *tert*-butanol capture ·OH, 10 mL H_2_O). (b) Fluorescence spectra of 2-hydroxy terephthalic acid (100 mL, 10 mg L^−1^ terephthalic acid solution, see supporting information for preparation method, excitation wavelength 310.4 nm, emission wavelength 426.4 nm). (c) Mass spectra of 4-vinyl benzoic acid (10 mL, 10 mmol L^−1^ PVBA). (d) ESR signals of ·H. (e) ESR signals of ·OH. (f) Schematic diagram of acoustic chemical decomposition of water.

To investigate the radical species generated during the experiment, *tert*-butanol and CCl_4_ were employed as trapping agents of ·OH and ·H [50,51], respectively. When 1 mL of *test*-butanol was added, the H_2_O_2_ generated by the system decreased from 245 μmol L^−1^ to 127 μmol L^−1^ in 40 min (Fig. S25a). When 1 mL of CCl_4_ was added, the H_2_ generated by the system decreased from 2.66 to 0.5 μmol h^−1^. The introduction of *tert*-butanol as the trapping agent for ·OH notably elevated the H_2_ yield (Fig. S25b). These outcomes conclusively indicate the presence of ·OH and ·H (Fig. 6a). Concurrently, quantitative analyses of ·OH under Ar and N_2_ atmosphere were conducted (Fig. 6b and Fig. S26). The results showed that the concentrations of ·OH were 7.9 and 5.4 μmol L^−1^ h^−1^ under Ar and N_2_, respectively (Fig. S26c). The molar concentration of ·OH was lower under N_2_, and thus the yield of H_2_O_2_ was lower than that under the Ar atmosphere [52]. ·H is capable of hydrogenating carbon–carbon double bonds [53,54]. For verification, 4-vinyl benzoic acid (PVBA) was utilized as the substrate (Fig. 6c), and hydrogenated PVBA, was detected at *m/z* of 149 by liquid chromatography-mass spectrometry (LC-MS), which was in good agreement with the predicted value of 149.0608. This result confirms the existence of ·H in the sonication process. To provide stronger evidence, we performed electron spin resonance (ESR) spin-trapping with a high concentration of 5,5-dimethyl-1pyrroline *N*-oxide (DMPO, 500 mM), which converts the radical intermediate into a long-lived spin adduct. The ESR spectroscopy displayed negligible signals without sonication. Intriguingly, after 30 min of sonication, nine ESR peaks with intensity ratios of 1 : 1 : 2 : 1 : 2 : 1 : 2 : 1 : 1 : 1 : 1 (Fig. 6d) and four ESR peaks with intensity ratios of 1 : 2 : 2 : 1 (Fig. 6e) appeared, while the ESR signals of O_2_^·−^ and ^1^O_2_ were negligible (Fig. S27), which are consistent with the spectral features of DMPO ·OH and DMPO ·H, respectively [55,56]. These findings further corroborate that the sonochemical process can decompose pure water into ·OH and ·H under the Ar atmosphere (Fig. 6f).

### Environmental implications

Ultrasound acts on water to generate cavitation bubbles, the collapse of which leads to the generation of the active species—·H, ·OH, H_2_O_2_, and HOO^−^. This research investigates the impacts of various experimental parameters, such as atmospheric conditions, ultrasonic power, pH, and temperature, on these processes. CFD simulations and experimental results show that Ar provides the optimal atmosphere condition, while extreme pH levels exert an inhibitory effect on the formation of H_2_O_2_. Additionally, high ultrasonic power and a water temperature of 20°C are found to be conducive for the formation of H_2_O_2_. Under optimal conditions, the rate of ultrasonic decomposition of water to produce H_2_O_2_ was as high as 351 μmol L^−1^ h^−1^. The ESR assay substantiated the generation of ·H and ·OH radicals through ultrasonic processes, and isotope labeling experiments confirmed that both the O atoms in the generated H_2_O_2_ and H atoms in the generated H_2_ were derived from H_2_O.

A comparison of the purity of H_2_O_2_ synthesized via various methods reveals that sonochemically generated H_2_O_2_ possesses extremely low proportion of organic by-products with the absence of metal ions, comparable to H_2_O_2_ synthesized through photochemical and electrochemical methods. More importantly, H_2_O_2_ synthesized by ultrasonic cavitation displayed super biosafety by *in vitro* cell and *in vivo* mouse experiments, compared with photocatalytic, electrocatalytic, and commercial H_2_O_2_ solutions. For the wound healing in medicine, the high-purity H_2_O_2_ would avoid the migration of organic or metallic impurities into the human body. It also has broad applications on water treatment and environmental disinfection, which could prevent the additionally transfer of impurities into atmosphere (especially indoor environment), natural waters, and soil ecosystem. In the future, this method could be improved upon the design of flow reactors, optimization of ultrasonic parameters, recycling inert gases, and coupling with renewable energy sources, which will make this method possess higher energy efficiency and stronger application prospects.

## CONCLUSIONS

This study unveils an infusive methodology for the efficient synthesis of H_2_O_2_ solution with high purity from H_2_O by ultrasonic cavitation with inert gases. The H_2_O_2_ solution synthesized by ultrasonic cavitation showed super biosafety by *in vitro* cell and *in vivo* mouse measurements. CFD simulations, isotopic labeling experiments, and radical detections proved that, sonication-induced local high temperature and pressure split pure water to ·OH and ·H radicals, which subsequently couple to generate H_2_O_2_ and H_2_. This work proposes an infusive methodology for the efficient synthesis of super-biosafety H_2_O_2_ solution without the addition of metals or organic reagents, and provides a theoretical foundation and expanded perspective for subsequent biological applications and research in ultrasonic chemistry.

## Supplementary Material

nwag218_Supplemental_File
