# Supplementary material for: Synthesis of super-biosafety hydrogen peroxide solution by ultrasonic cavitation
Source: Natl Sci Rev. 2026 Apr 9;13(13):nwag218. doi: 10.1093/nsr/nwag218 (PMC13386504; doi:10.1093/nsr/nwag218)
Supplement: nwag218_Supplemental_File [file nwag218_supplemental_file.pdf]

Supporting Information

Synthesis of Super Biosafety Hydrogen Peroxide Solution by Ultrasonic Cavitation

Qiao Wang[a#], Feng Hong[b#], Xuan Xia[c#], Di Huang*[a,b], Li Wang[b], Decheng Wang*[c], Hongwei Huang*[d], Xin Ying Kong[e], Yee Wen Teh[e], Minghui Lv[a], Tao Gao[a], Yingping Huang[b], Liqun Ye*[a,b]

[a] Q. Wang, D. Huang, T. Gao, M. H. Lv. L. Q. Ye

College of Materials and Chemical Engineering, Key Laboratory of Inorganic Nonmetallic Crystalline and Energy Conversion Materials, China Three Gorges University, Yichang 443002, China.

E-mail: lqye@ctgu.edu.cn (L. Q. Ye)

E-mail: huangdi@ctgu.edu.cn (D. Huang)

[b] F.Hong, D. Huang, L. Wang, Y. P. Huang

Engineering Research Center of Eco-environment in Three Gorges Reservoir Region, Ministry of Education, China Three Gorges University, Yichang 443002, Hubei (P. R. China)

[c] Dr. D. C. Wang,

Hubei Key Laboratory of Tumor Microenvironment and Immunotherapy, College of Basic Medical Sciences, China Three Gorges University, Yichang 443002, P.R China

E-mail: dcwang99@163.com (D. C. Huang)

[d] Dr. H. W. Huang

Beijing Key Laboratory of Materials Utilization of Nonmetallic Minerals and Solid Wastes, School of Materials Science and Technology, China University of Geosciences (Beijing) Beijing 100083, P. R. China

E-mail: hhw@cugb.edu.cn (H. W. Huang)

[e] Dr. X. Y. Kong

School of Chemistry, Chemical Engineering and Biotechnology, Nanyang Technological University, Singapore, 21 Nanyang Link, 637371 Singapore.

Section 1. Experimental Procedures

1.1 Chemicals

Sodium hydroxide (NaOH), tert-butanol, and terephthalic acid (TA) were purchased from China Aladdin Co. Ltd, carbon tetrachloride (CCl_4), 4-carboxyphenylboronic acid, lumefantrine, and benzoic acid were procured from Shanghai Macklin Co. Ltd., 30% hydrogen peroxide (30% H_2O_2) was supplied by Zhejiang Thermo Pharmaceuticals, and sulfuric acid (H_2SO_4) was supplied by Cologne Co.

1.2 Ultrasonic production of H_2O_2

Ultrasonic generation of H_2O_2 was carried out in a 50 mL airtight glass vial in a 40 kHz,80 W SB-80 ultrasonic cleaner supplied by Ningbo Xinzhi Bio-Tech Co. In a typical experiment, 10 mL of deionized water (pH ≈ 6.5) was used, and a cooled circulating water bath was used to maintain the temperature at 20 °C. The samples were collected every 10 min. Typically, ultrasonic generation of H_2O_2 was carried out in an Ar atmosphere, unless otherwise stated.

1.3 The detection method for H_2O_2

Phosphate buffer was prepared by dissolving 2.8756 g of $K_2HPO_4{\cdot}3H_2O$ and 11.935 g of KH_2PO_4 in 200 mL of deionized water. Following that, N, N-diethyl-1,4-phenylenediamine sulfate (DPD, 97%, Aladdin), and peroxidase (POD, horseradish, Aladdin) stock solutions were prepared dissolving 0.1 g of DPD in 10 mL of 0.05 M H_2SO_4 solution and 10 mg of POD in 10 mL of deionized water, respectively. Throughout the experiment, 2.5 mL of the solution to be tested was added to the quartz tube, followed by adding 0.4 mL of phosphate buffer, 50 μL of POD solution, and 50 μL of DPD solution sequentially, and the resulting mixture was mixed well. The absorbance at 552 nm was measured by UV-2600 (Shanghai Tianmei Scientific Instrument Co.). The H_2O_2 concentration was calibrated by diluting 30% of the H_2O_2 stock solution to prepare a standard curve (Figure S2).

1.4 Taking chemiluminescent photographs

Luminescence photographs were taken with an iPhone (Apple brand), and the experiments were performed during sonication in 50 mL of luminal solution (100 $mmol{\cdot}L^{-1}$ NaOH and 2 $mmol{\cdot}L^{-1}$ luminal) to compare the strength of the cavitation field by the passage of Ar and N_2.

1.5 Photocatalytic degradation of RhB

In a typical experiment, 0.5 mL salutation (including H_2O, commercial H_2O_2, H_2O_2 prepared by the acoustic method, H_2O_2 prepared by the photocatalytic method, H_2O_2 prepared by electrocatalytic method) and 3.5 mL of $5mg{\cdot}L^{-1}$ RhB was taken in the cuvette. The solution was

placed under an ultraviolet lamp (253 nm) and the absorbance of the solution was measured after 5h of reaction.

1.6 Quantitative testing of •OH

0.04 g of terephthalic acid (TA) and 0.04 g of NaOH was configured into a solution of 500 mL. •OH will generate 2-hydroxy terephthalic acid (HTA) with terephthalic acid (TA), which was quantified by fluorescence emission spectrometry (SHIMADZU, RF-6000A) with an excitation wavelength of 315 nm and an emission wavelength of 425 nm.

1.7 Testing of •H

10 mmol L^{-1} of PVBA solution was prepared and sonicated for 10 min, and a control experiment was set up by preparing 10 mmol·L^{-1} of PVBA solution without sonication. The peak position was detected by SHIMADZU-LC20A, LCMS-8050 triple quadrupole liquid mass spectrometer (Shimadzu).

1.8 Isotopic labeling

^{18}O isotope was tested on SHIMADZU-LC20A, LCMS-8050 triple quadrupole liquid mass spectrometer (Shimadzu). The sample was prepared with 4 ml of $H_2{}^{18}O$ and sonicated under argon gas for 20 min for 1 h to obtain $H_2{}^{18}O_2$, and 4 ml of H_2O was prepared as a comparison sample and sonicated under the same conditions for 1 h to obtain H_2O_2. 100 μmol·L^{-1} 4-carboxyphenylboronic acid was prepared, and 200 μL of 4-carboxyphenylboronic acid was taken to mix with 200 μL of $H_2{}^{18}O_2$ and H_2O_2, respectively, and allowed to stand for 20 min. The samples to be measured were injected into the microchannel and analyzed using liquid chromatography-mass spectrometry in negative ion mode to compare the intensity changes corresponding to 4-carboxy phenol at 137.1 m/z and 139.1 m/z. The experiment was carried out by replacing 10 mL of H_2O with D_2O, and the gas in the sealed bottle was detected in PM-QMS after sonication for 1 h. The peak at m/z=4 was observed.

1.9 ESR Testing

Electron series resonance (ESR) spectra under sonication conditions were recorded with a Deutsche-Bruker-A300. Under the Ar atmosphere, •H and •OH were detected before and after 30 min of sonication, respectively, using DMPO as a spin-trapping reagent.

1.10 Preparation of photocatalytic hydrogen peroxide

According to the literature,[1] synthetic resin material and 50 mg of catalyst were dissolved in 20 mL of water, followed by exposure to light at λ>400 nm (3 sunlight conditions) for 4h. The concentration of H_2O_2 synthesized was tested and formulated to about 1 m mol·L^{-1}.

1.11 Preparation of electrocatalyzed H_2O_2

For the preparation of the working electrode, 5 mg of carbon black was dispersed in a mixture of 780 μL of ethanol, 200 μL of water, and 40 μL of naphthol to form a slurry. The slurry was applied slowly and dropwise to a carbon paper with a length and width of 1.5 cm×1.5 cm as the working electrode. The loading amount of the carbon black on the carbon paper was made to be 1.5 mg cm^2. For a standard test, a standard three-electrode system was formed using carbon paper loaded with carbon black as the working electrode, Hg/HgO as the reference electrode, and carbon rods as the counter electrode in an oxygen-saturated 0.1 $mol \cdot L^{-1}$ KOH solution for electrochemical testing. The i-t curves were performed at a potential of 0.4 V vs. RHE for 5 h. At the end of the test, hydrogen peroxide was obtained from the working electrode on the side of the H-type electrolyzer. The H_2O_2 generated was obtained from the site where the working electrode was located. The generated H_2O_2 was prepared to a concentration of about 1 $mmol \cdot L^{-1}$.

1.12 *In vitro* testing

(1) Cell culture.

Human lung adenocarcinoma cell line A549 cells were cultured in Dulbecco's Modified Eagle's Medium (DMEM) (Boster, PYG0070) containing 100 IU/mL penicillin and 100 μg/mL streptomycin, supplemented with 10% fetal bovine serum (Capricorn, FBS-LE-12A). The cells were incubated in a humidified 37°C incubator with 5% CO_2. When the cells reached 80% to 90% confluence, they were seeded onto culture plates as needed.

(2) CCK-8 Assay for Cell Viability

A549 cells were seeded at a density of 8×10^3 cells per well in a 96-well plate. After overnight incubation, the cells were treated with hydrogen peroxide prepared using different methods (photocatalysis, electrocatalysis, sonication, commercial, Cds) at a concentration of 250 μM for 24 and 29 hours, with PBS as the control. Subsequently, the CCK-8 reagent (GLPBIO, GK10001) was mixed with DMEM in the ratio of 1:10, and 110 μL of the mixture was added to each well of the 96-well plate. The culture plate was then incubated in a cell incubator for 30 minutes, and the absorbance at 450 nm was measured for each well. Cell viability was calculated according to the formula provided in the reagent manual.

(3) Flow Cytometry for Detection of Cell Apoptosis

A549 cells were seeded at a density of 5×10^5 cells per well in a 6-well plate. After overnight incubation, the cells were treated with hydrogen peroxide prepared using different methods (photocatalysis, electrocatalysis, sonication, commercial, Cds) at a concentration of 250 μM for 24 hours, with PBS as the control. Subsequently, all cells from each treated well were collected. The cell pellets obtained after centrifugation were resuspended and washed once with sterile

PBS. The cell pellets obtained after the second centrifugation were resuspended in a binding buffer, followed by the addition of Annexin V-FITC and PI staining solution (Beyotime, C1062M). The mixture was gently vortexed and incubated in the dark for 15 minutes before flow cytometry analysis for cell apoptosis. The results were analyzed using Flowjo 10.8.1 software.

1.13 *In vivo* testing

(1) Animals and Experimental Design

To test the toxicity and safety of ultrapure H_2O_2, specific pathogen-free (SPF) C57BL/6J mice (eight-week-old, male) fed with chow diet (10 kcal%, Diet: D12450B; Beijing HFK Bioscience Co Ltd, China) were divided into three groups: (1) ultrapure H_2O_2 (H_2O_2-U) group (n=10): These mice were subjected to give H_2O_2-U by gavage (dissolved in double-distilled water, 500 μmol/L). The dosage was 5μL/g (body weight) per day for the first 10 days, up-titrated to double dosage every 10 days; (2) commercial H_2O_2 (H_2O_2-C) group (n=10): These mice were subjected to give commercial H_2O_2 (purchased from Zhejiang Thermo Pharmaceuticals, 500 μmol/L, dissolved in double-distilled water) by gavage. The dosage (volume) was the same as the H_2O_2-U group; (3) control group (n=10): These mice were given double-distilled water (equal volume to the other two groups) by gavage; After being treated for 30 days, all mice were anesthetized by inhalational isoflurane and blood were collected by eyeball enucleation. The related organs were weighed, fixed in 4% paraformaldehyde or snap frozen in liquid nitrogen, and stored in a -80℃ refrigerator.

In this study above, C57BL/6J mice were purchased from the Laboratory Animal Center of China Three Gorge University and then housed in the SPF-grade environment (23±2℃, 55%±10% humidity, a 12-h light/black cycle) in the Animal Center of China Three Gorges University. Animal studies were reported in compliance with the ARRIVE guidelines and carried out in accordance with the National Institutes of Health guide for the care and use of Laboratory animals (NIH Publications No. 8023, revised 1978). The animal experiments were reviewed and approved by the Animal Care and Use Committee of China Three Gorges University (2024010U3).

(2) Hematoxylin-eosin staining and Masson's trichrome stain.

For Hematoxylin-eosin (H&E) staining, the samples (stomach, jejunum, colon) were fixed in 4% paraformaldehyde for 48 h, and transferred to 75% ethanol. And then the samples were embedded in paraffin. The 5 μm serial sectionswere prepared and stained with a hematoxylin-eosin solution according to the manufacturer's instructions. The detailed protocol was seen in our previous publications. [2]

Fibrosis is a pathological process in response to chronic tissue injury or chronic inflammation that is characterized by excessive accumulation of collagen.[3,4] To compare the fibrosis situation under different treatments, we evaluate the fibrosis of organ injury by Masson's trichrome staining. First, 5μm sections were prepared, deparaffinized, and hydrated in distilled water. Bouin's fixative was then used as a mordant at 56°C for 1 h. The formalin-fixed, paraffin-embedded sections were cooled and washed in running water until the yellow coloring disappeared. The samples were stained in Weigert's hematoxylin stain for 10 min, thoroughly washed in tap water for 10 min, stained again in an acid fuchsin solution for 15 min, and rinsed in distilled water for 3 min. After rinsing, the slides were treated with phosphomolybdic acid solution for 10 min and then rinsed in distilled water for 10 min. Finally, slides were stained with a light-green solution for 2 min and rinsed in distilled water. After thorough dehydration using alcohol, the slides were mounted, and coverslips were placed onto them. The intensity of fibrosis-positive staining in tissues was observed by Olympus BX63 microscopy (10 or 20× magnification). The detailed protocol was seen in our previous publications.[5]

(3) TUNEL staining

The main staining steps were as follows: the juvenile fish were fixed in 3 % paraformaldehyde (3% PFA) and stored at room temperature for 4 h. The organs (stomach, colon) were dehydrated with methanol and 1× PBST in a gradient of 100% PBST, 25 % MeOH in PBST, 50 % MeOH in PBST, 75 % MeOH in PBST, and 100% MeOH for 5 min each. The gradient was rehydrated on day 2 by inverting the gradient of the dehydration volume ratio. The sample was washed with 500μl of 20μg/L Protease K to ensure that the sample was submerged, and the reaction was carried out at 37°C for 60 min and then washed with 1× PBST for 5 min each time. After the reaction at 37 °C for 60 min, the samples were washed 3–5 times with 1× PBST for 5 min each time, then treated with 3 % PFA for 20 min to inactivate Protease K, and washed 5 times with 1× PBST for 5 min each time. 2 μL of TdT enzyme in a TUNEL kit (Servicebio Technology Co., China) and 48μL of photolabeling solution were taken and thoroughly mixed as an enzyme reaction solution(37°C,1h). The nucleus was stained at room temperature for 10 min by DAPI. Finally, the section was photographed by confocal microscopy (Olympus BX53, Japan). The area of green fluorescence was the target cell of apoptosis.

(4) Biochemical analysis

The detection of serum alanine aminotransferase (ALT), and aspartate aminotransferase (AST) levels was carried out by enzymatic method according to the direction of the commercial kit (Nanjing Jiancheng Co, C009-2-1, C010-2-1) to evaluate the liver function. The detection

of serum urea nitrogen (BUN), and creatinine (sCr) levels was carried out by enzymatic method according to the direction of the commercial kit (Nanjing Jiancheng Co, C013-2-1, C011-2-1) to evaluate the liver function. All test above was carried out by enzyme method and spectrophotometry.

Section 2 Numerical setup for the CFD model:

The computational fluid dynamics (CFD) tool was applied to obtain an in-depth understanding of the physical effects of bubble collapse under an ultrasonic environment. In general, the whole process of CFD simulation contains flow domain establishment, mesh generation, solution, and post-processing. The whole computational assembly is depicted in Supplementary Fig.3, where the detail of the mesh resolution is also presented. A cavitation bubble was assumed in the center of the liquid part, and an oscillating pressure field using a single-frequency sound wave (*f*=40 kHz) was defined to model the ultrasonic condition. The computations in the present work were performed based on the platform ANSYS Fluent R2022. The bubble was patched in the center of the liquid region using the CFD code. The scheme of pressure-implicit with the splitting of operators (PISO) was assigned for the coupling between velocity and pressure. The pressure staggering option (PRESTO!) was used for the pressure discretization algorithm, while the second-order upwind scheme was adopted for the terms of density, turbulent kinetic energy, momentum, and energy. The compressive method was selected for the transport equation of vapor volume fraction, and a first-order implicit scheme was adopted for the transient formulation to calculate the time integrations. The numerical methodology includes the solving of the continuity and Navier-Stokes equations, as well as the application of the volume of fluid (VOF) model, which respectively reads,

$$\nabla \cdot \vec{u} = 0 \tag{1}$$

$$\frac{\partial(\rho\vec{u})}{\partial t} + \nabla \cdot (\rho\vec{u}\vec{u}) = -\nabla p + \nabla \cdot \left\{\mu[\nabla\vec{u} + (\nabla\vec{u})^T]\right\} + \rho\vec{g} + F_s \tag{2}$$

$$\frac{\partial\alpha_k}{\partial t} + \vec{u}_k \cdot \nabla\alpha_k = 0 \tag{3}$$

$$\sum_{k=1}^{2}\alpha_k = 1 \tag{4}$$

$$\rho(\vec{x},t) = \alpha(\vec{x},t)\rho_l + (1-\alpha(\vec{x},t))\rho_g \tag{5}$$

$$\mu(\vec{x},t) = \alpha(\vec{x},t)\mu_l + (1-\alpha(\vec{x},t))\mu_g \tag{6}$$

where ρ, u, p, μ, and α denote the density, velocity, pressure, dynamic viscosity, and volume fraction, respectively. The subscript k=1 represents the liquid phase, while k=2 represents the gas phase. F_s is the surface tension force per unit volume for the mixture phase.

Section 3 Model validation

To validate the numerical model, the Volume of Fluid (VOF) multiphase model is employed coupled with the same computational algorithms for density, turbulent kinetic energy, momentum, and energy terms. A bubble with a radius of 0.2 mm is positioned in a square computational domain of 1 mm length, filled with water vapor and located near the bottom wall. A constant wall pressure of 0.1 MPa is applied to the computational domain, aligning with the experimental work of V. Minsier (*J. Appl. Phys.*, 2009, 106, 084906). Due to the milder wall pressure conditions, the bubble exhibits a significantly extended evolutionary period. The predicted bubble shape and size demonstrate excellent agreement with the visualized images reported in V. Minsier's work, confirming the model's accuracy as shown in the below figure.

On the other side, the flow turbulence is also considered in the CFD simulations, since the Realizable k-epsilon turbulence model has been adopted and the fluid disturbance is modeled by this turbulence closure. The net effect of fluid disturbance on the mean flow is predicted through two additional transport equations (ANSYS, Inc. ANSYS Fluent Theory Guide: Release 2022 R2 [EB/OL]. Canonsburg: PA 15317):

$$\frac{\partial}{\partial t}(\rho k)+\frac{\partial}{\partial x_j}(\rho k u_j)=\frac{\partial}{\partial x_j}\left[\left(\mu+\frac{\mu_t}{\sigma_k}\right)\frac{\partial k}{\partial x_j}\right]+G_k+G_b-\rho\varepsilon-Y_M+S_k$$

$$\frac{\partial}{\partial t}(\rho \varepsilon)+\frac{\partial}{\partial x_j}(\rho \varepsilon u_j)=\frac{\partial}{\partial x_j}\left[\left(\mu+\frac{\mu_t}{\sigma_\varepsilon}\right)\frac{\partial \varepsilon}{\partial x_j}\right]+\rho C_1 S\varepsilon-\rho C_2\frac{\varepsilon^2}{k+\sqrt{\nu\varepsilon}}+C_{1\varepsilon}\frac{\varepsilon}{k}C_{3\varepsilon}G_b+S_\varepsilon$$

where all the parameters in the equations can be referred in "ANSYS Fluent Theory. Guide".

Section 3. Supporting Figures

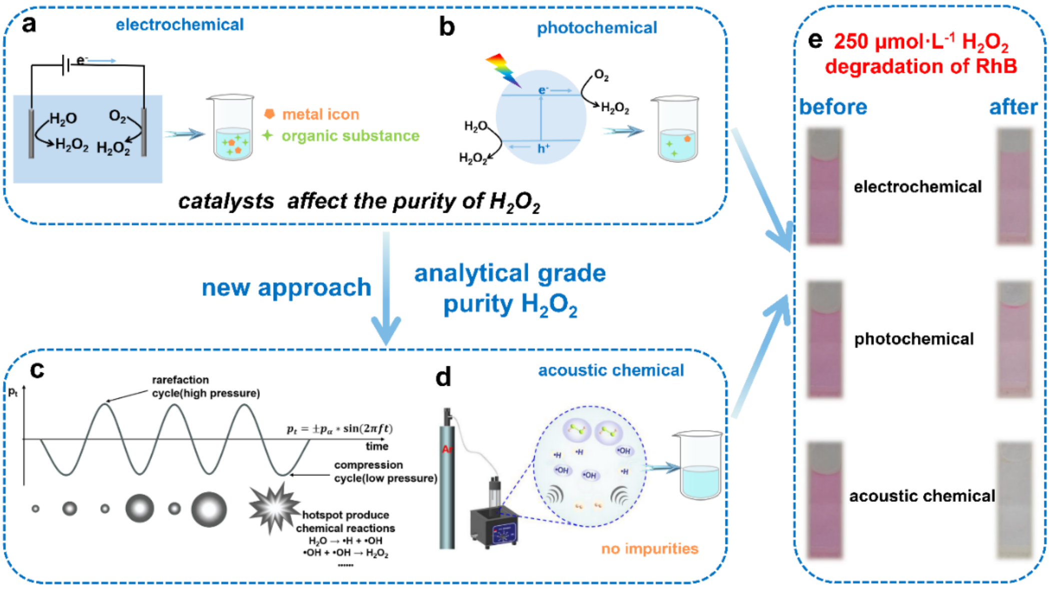

Figure S1. Different methods of preparing H_2O_2. a) Electrochemical preparation of H_2O_2, b) Photochemical preparation of H_2O_2, c) The growth state of cavitation bubbles with changes in ultrasonic pressure, d) Schematic diagram of the setup for the sonochemical preparation of H_2O_2, e) H_2O_2 generated by different preparation methods degrades RhB.

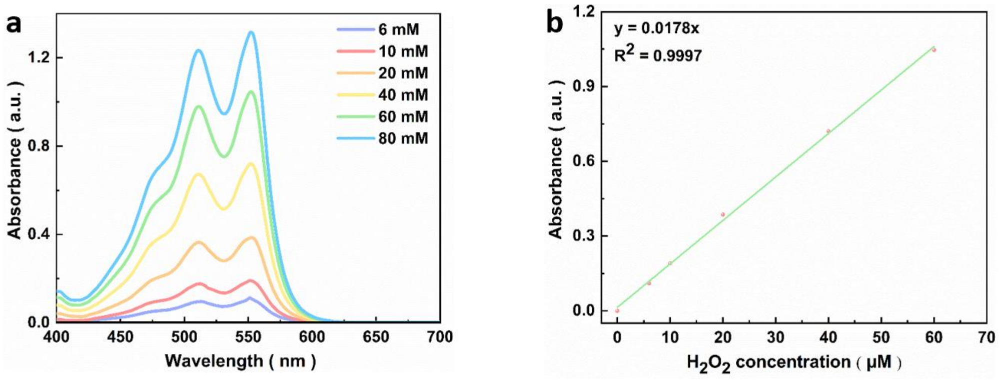

Figure S2. Different concentrations of H_2O_2 (0 μmol·L^{-1}, 6 μmol·L^{-1}, 10 μmol·L^{-1}, 20 μmol·L^{-1}, 40 μmol·L^{-1}, 60 μmol·L^{-1}, 80 μmol·L^{-1}) at wavelengths of 400~700 nm. (c) The standard curve of H_2O_2 concentration-absorbance was plotted according to b.

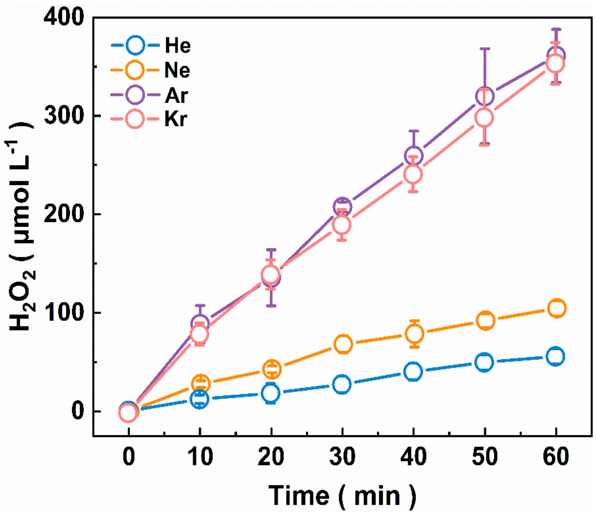

Figure S3. H_2O_2 production under the He, Ne, Ar and Kr atmospheric conditions (10 mL H_2O, f=40 kHz, P=2.77 W cm^{-2}).

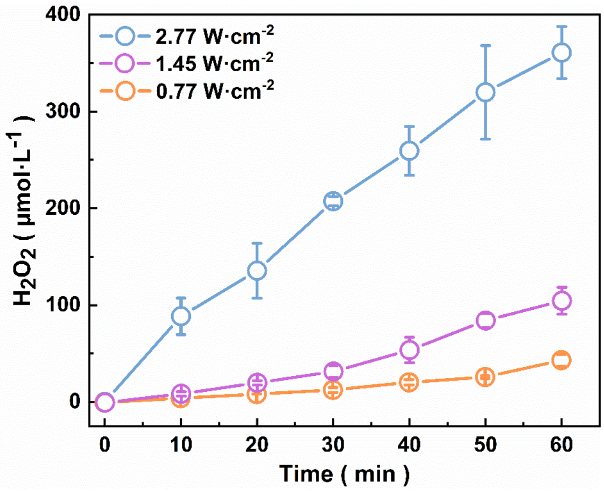

Figure S4. Effect of acoustic power on the rate of H_2O_2 yield (Ar,10 mL H_2O, temperature=20℃, pH=7.0, f=40 kHz).

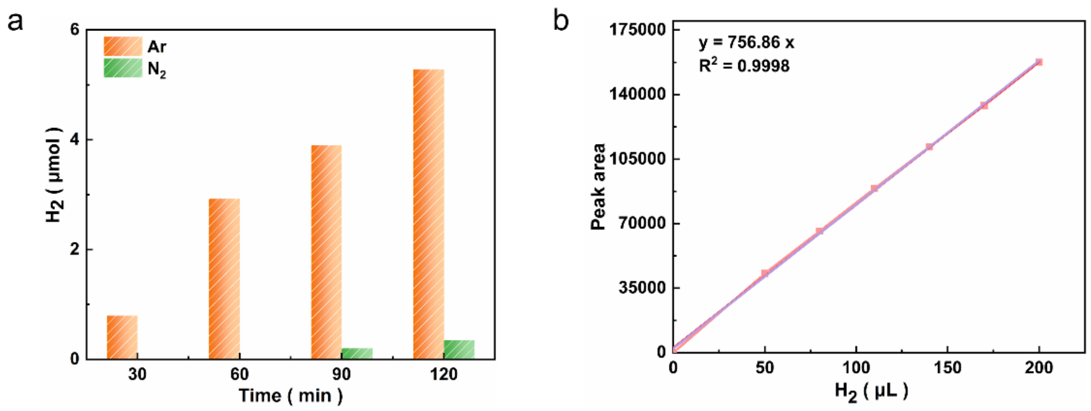

Figure S5. (a) Moles of H_2 in N_2 and Ar atmospheres. (b) H_2 volume-peak area standard curve.

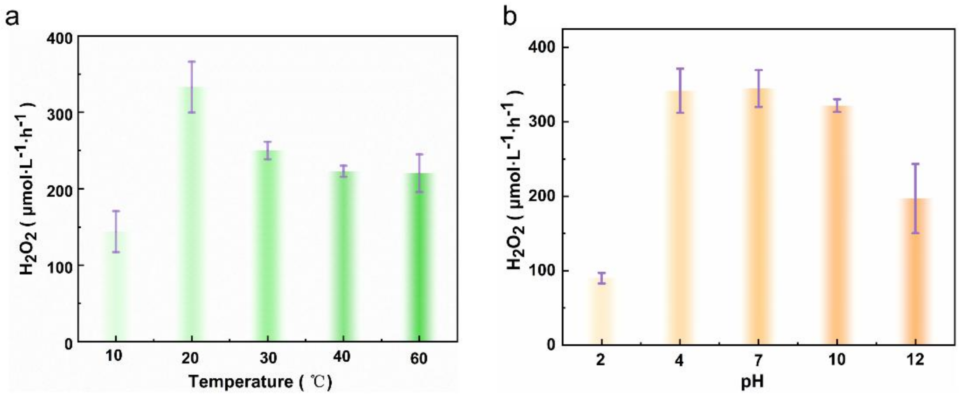

Figure S6. (a,b) Effect of different conditions on the rate of H_2O_2 generation: temperature of water, pH.

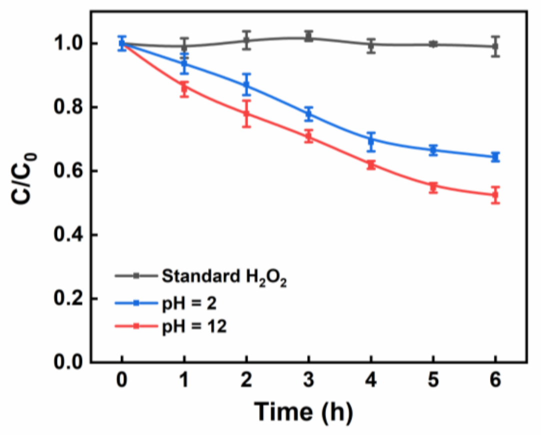

Figure S7. Concentrations changes of H_2O_2 under the acidic, alkaline, and near neutral conditions.

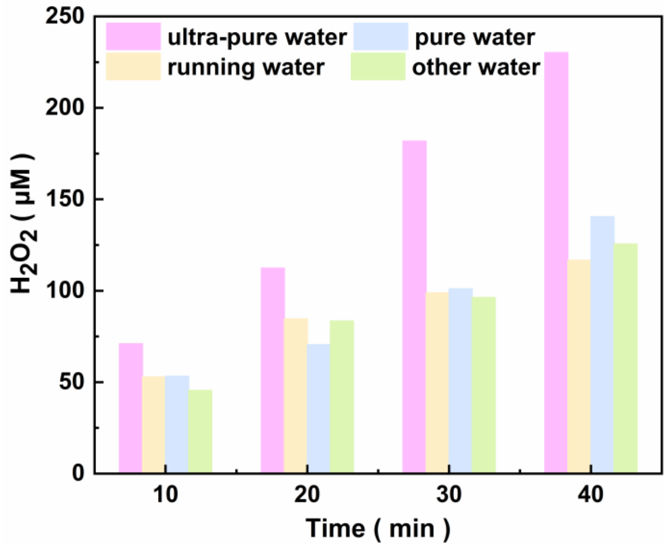

Figure S8. Acoustic catalyzed generation of H_2O_2 from different water qualities.

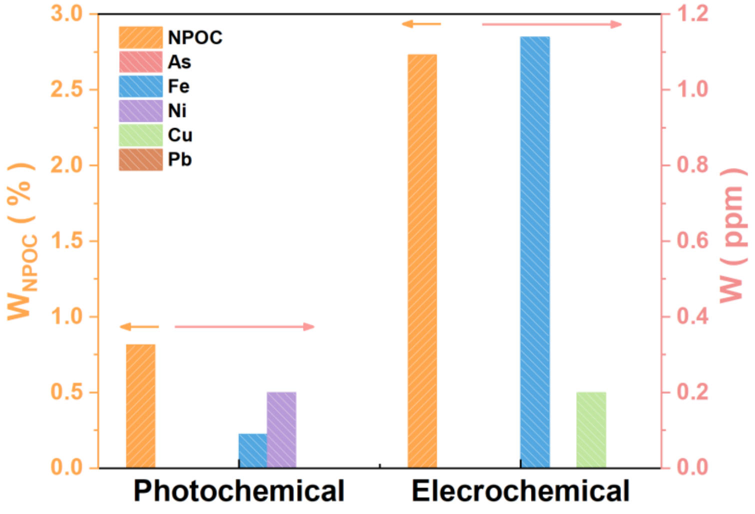

Figure S9. Mass fraction of NPOC and metal ions (As, Fe, Ni, Cu, and Pb) mass fraction in H_2O_2 synthesized by photochemical (H_2O_2-P) and electrochemical (H_2O_2-E) methods.

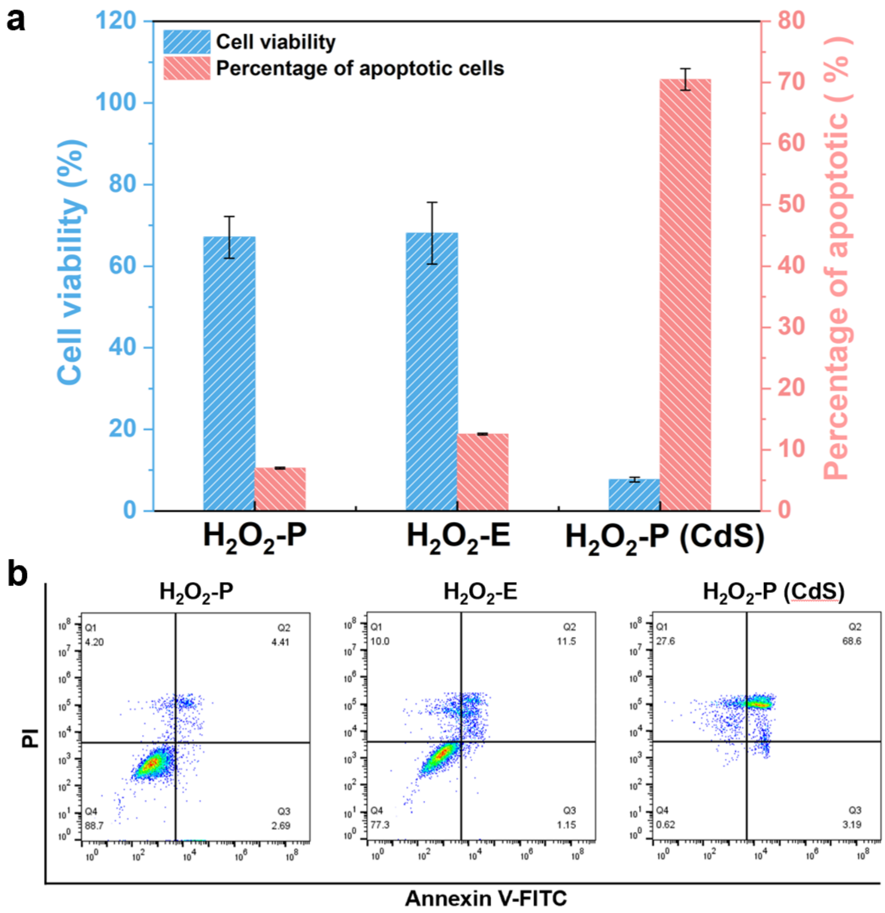

Figure S10. The biosafety measurements of H_2O_2 prepared by photocatalysis with non-metallic materials (H_2O_2-P), photocatalysis with metallic materials (H_2O_2-P (CdS)), and electrocatalysis (H_2O_2-E) *in vitro* cell experiments. (a) The cell viability of A549, and the percentage of apoptosis in A549 cells treated by H_2O_2 prepared using different methods (photocatalysis with non-metallic materials, electrocatalysis, and photocatalysis with metallic materials CdS) at a concentration of 250 μM for 24 h, with phosphate buffered saline (PBS) as the control. The photocatalysis with metallic materials CdS sample only treated 6 h. (b) The apoptosis in A549 cells treated by H_2O_2 prepared using different methods. The data were assessed using Annexin V-FITC/PI dual staining. Q1: Annexin V^-/PI^+, necrotic or mechanically damaged cells; Q2: Annexin V^+/PI^+, late apoptotic/necrotic cells; Q3: Annexin V^+/PI^-, early apoptotic cells; Q4: Annexin V^-/PI^-, viable cells.

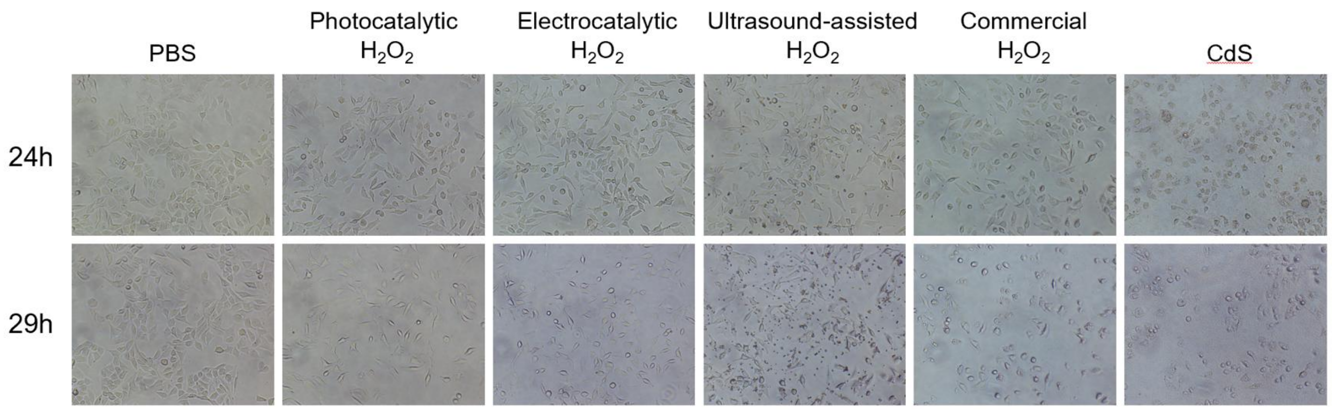

Figure S11. The morphology of A549 cell viability after treating by hydrogen peroxide prepared using different methods (photocatalysis, electrocatalysis, sonication, commercial, CdS) at a concentration of 250 μM for 24 and 29 hours, with phosphate buffered saline (PBS) as the control.

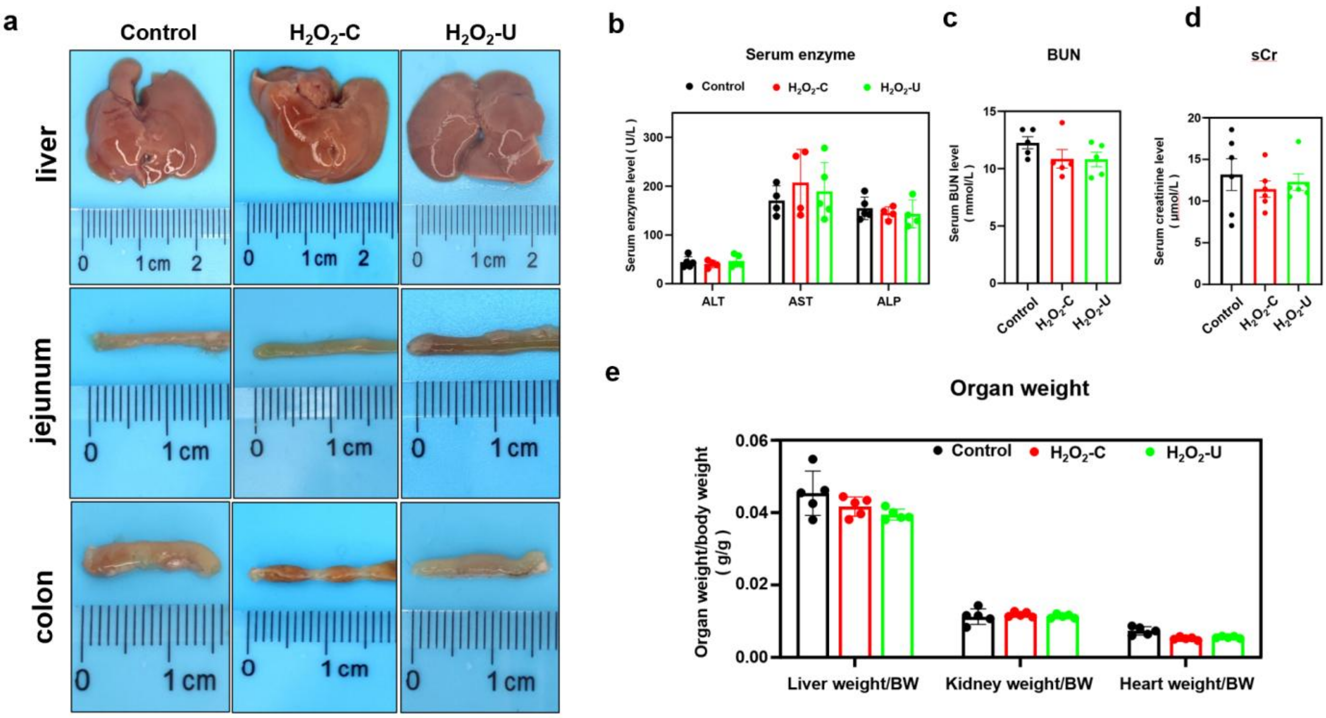

Figure S12. (a) Gross observation of liver, jejunum, and colon of C57BL/6J mice treated by H_2O_2-U, H_2O_2-C, and vehicle (double distilled water) by gavage and sacrificed at the end of experiments. (b) Serum liver enzyme level of ALT (alanine aminotransferase), AST (aspartate aminotransferase), ALP (alkaline phosphatase) (indicating the liver function) in the three groups above. (c) Serum BUN (blood urea nitrogen) and SCr (serum creatinine) level (indicating the renal function) in the three groups above. (d) The ratio of organ weight (liver, kidney, heart) and body weight. BW: body weight.

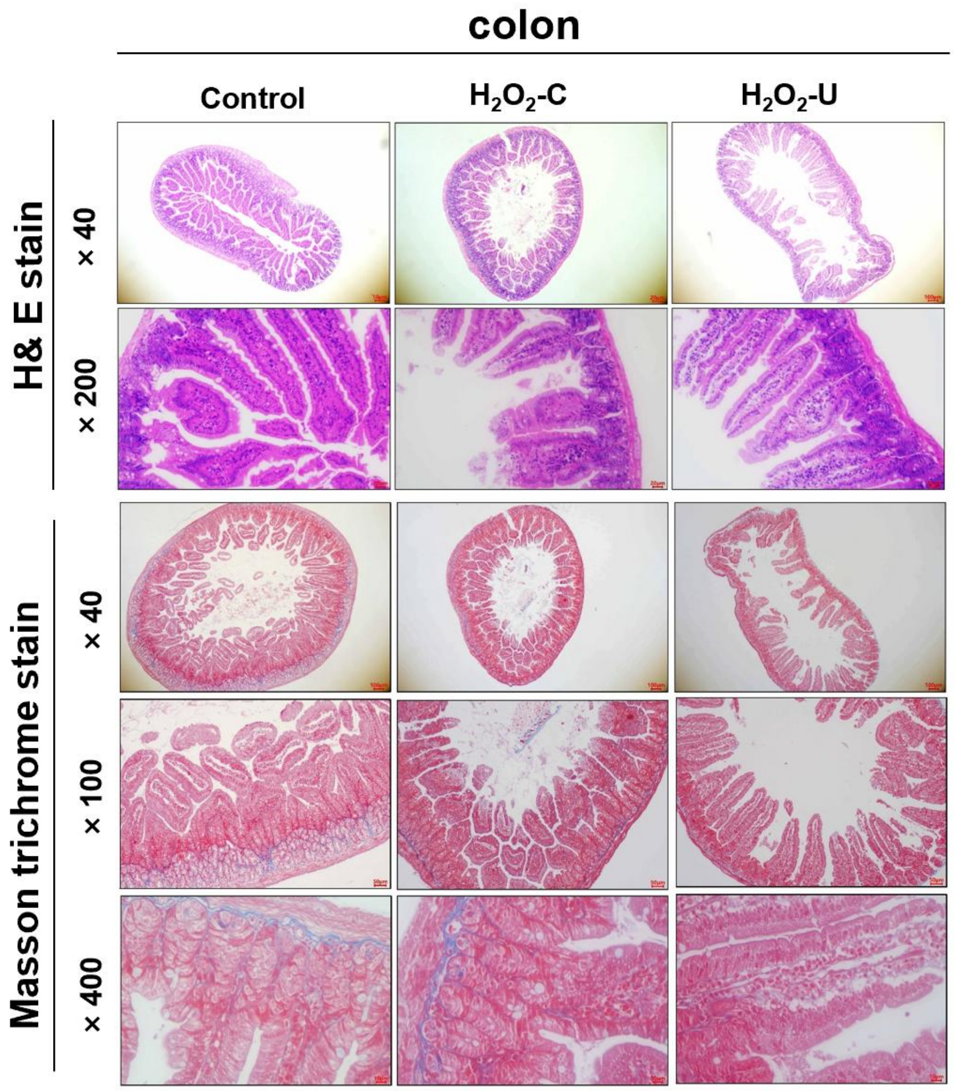

Figure S13. The morphological alteration of a transverse section of the colon.

The H&E stain(upper) and Masson trichrome stain (lower) of the colon of C57BL/6J mice treated by H_2O_2-U, H_2O_2-C, and vehicle by gavage and sacrificed at the end of experiments. By H&E stain, there was atrophy and thin mucosa of the colon while the enteric cavity became enlarged in two H_2O_2 groups compared with the vehicle group. Meanwhile, there were ruptures of small finger-like villi which became sparse in two H_2O_2 groups compared with the vehicle group. However, there were no obvious differences in colon injury between H_2O_2-U and H_2O_2-C groups. By Masson trichrome stain, there were no significant differences in fibrosis of intercellular interstitium between the three groups.

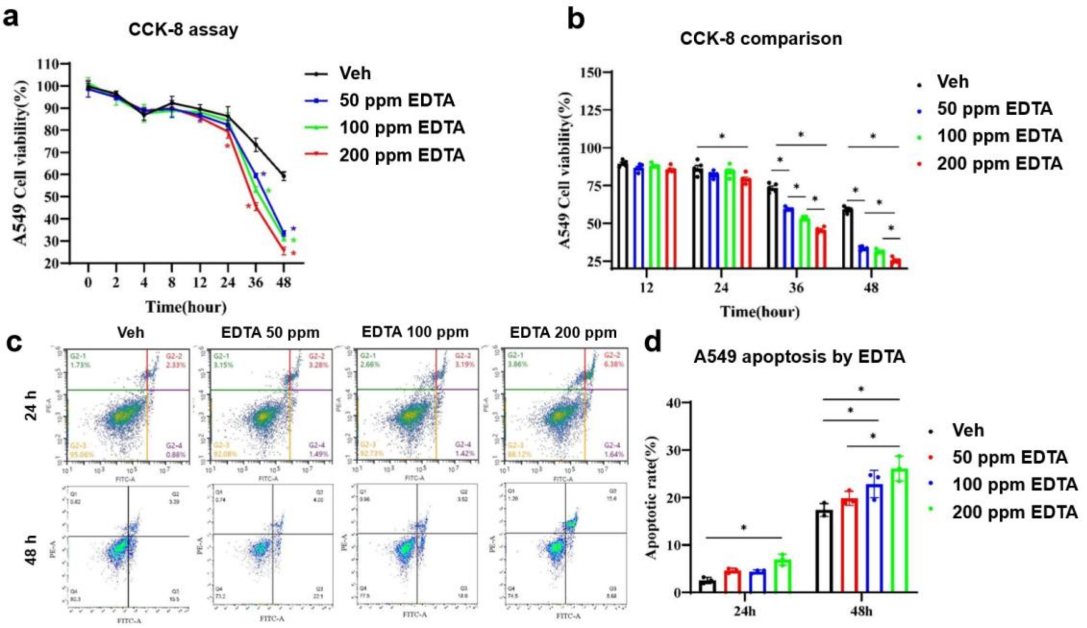

Figure S14. Cell viability was evaluated by CCK-8 assay and apoptosis that A549 cell was treated by EDTA in indicated time-course and concentration-courses.

(a) The cell viability was not altered in duration less than 12 hours. at 24 hour or later, there was different between groups in 12, 24, 36, 48h. *, P<0.05, compared with Veh (Vehicle) group in the indicated timepoint. (b) The Bar graph to show the statistical comparison between intergroups in 12, 24, 36, 48 hours. *, P<0.05, compared with intergroups. (c) Apoptotic rate of A549 treated by EDTA for 24, 48h hours. Flow cytometry method (Annexin V-FITC stain apoptosis kit, Beyotime, Hangzhou) to check apoptosis in A549 cells treated by EDTA in different concentrations (50, 100, 200 ppm). The data were assessed using Annexin V-FITC/PI dual staining. Q1: Annexin V^-/PI^+, necrotic or mechanically damaged cells; Q2: Annexin V^+/PI^+, late apoptotic/necrotic cells; Q3: Annexin V^+/PI^-, early apoptotic cells; Q4: Annexin V^-/PI^-, viable cells. (d) The apoptotic rate was checked by Annexin V/FITC and flow cytometry. A549 cell was treated by EDTA of different concentrations in 24 hours or 48 hours(C). These assays were carried by three dependent times. The bar graph was statistically analyzed and shown the difference (D). *, P<0.05, compared with intergroups.

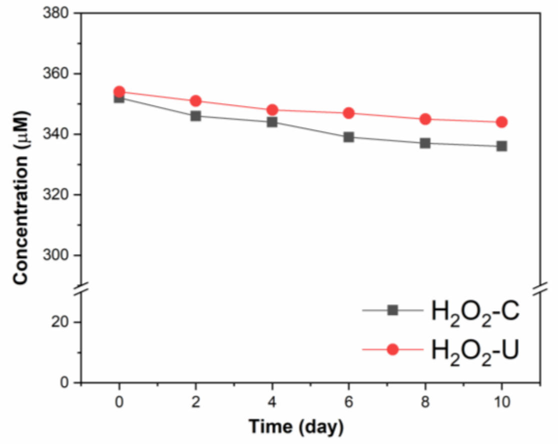

Figure S15. Concentrations of commercial H_2O_2 (H_2O_2-C, black line) and H_2O_2 synthesized by ultrasonic cavitation (H_2O_2-U, red line) during the 10 days long-term storage.

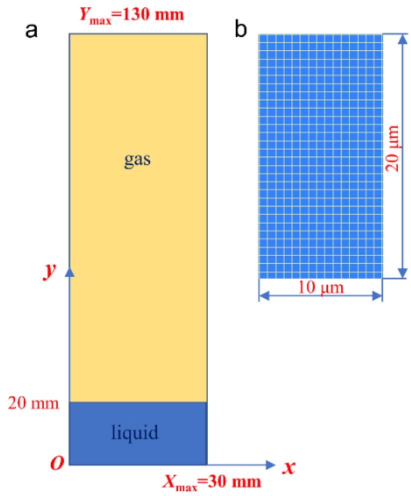

Figure S16. Physical model of present CFD calculation. (a) Computational domain and (b) mesh resolution.

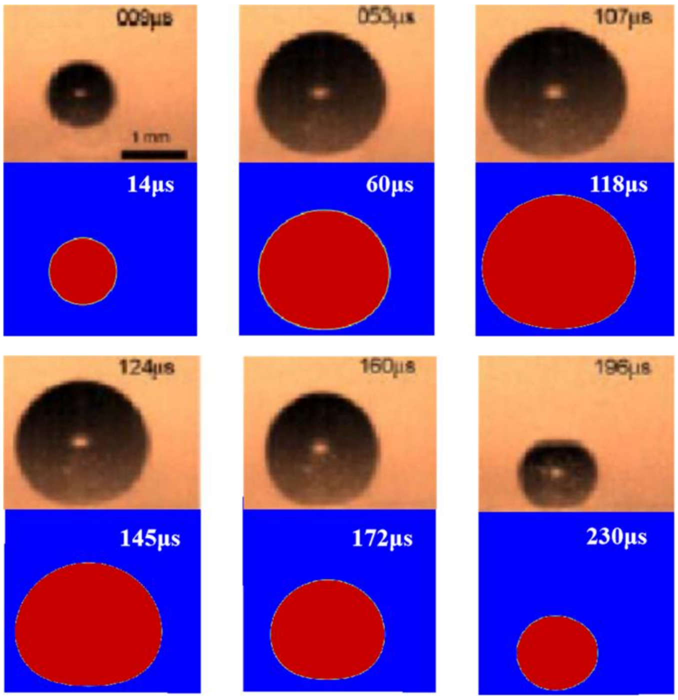

Figure S17. Comparisons of a bubble evolution by CFD simulation and available experimental figures.

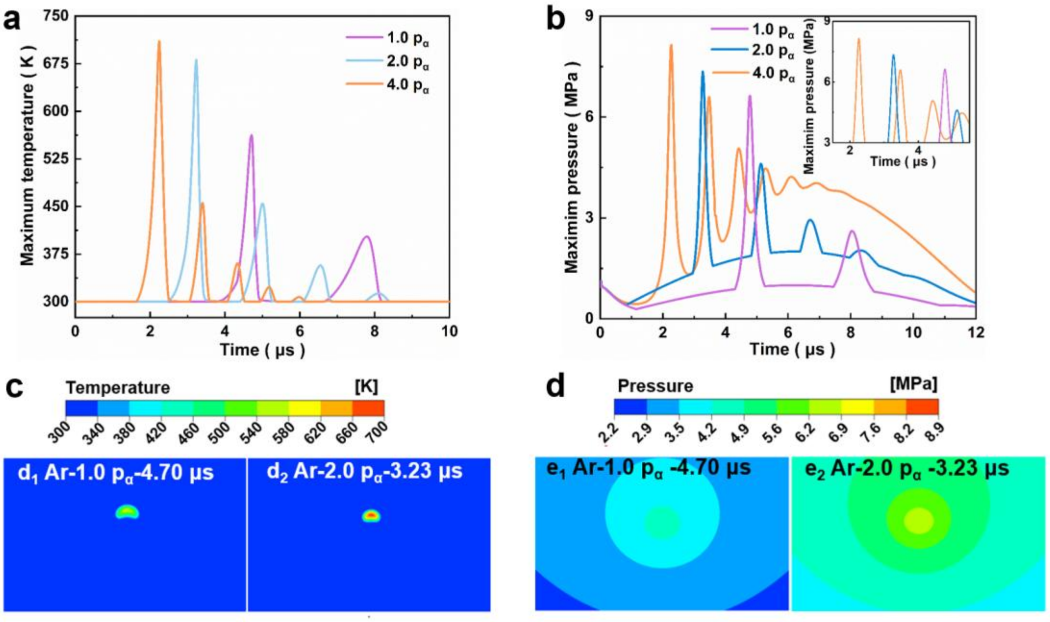

Figure S18. (a) Influences of pressure amplitude on the time-dependent local maximum temperature for Ar atmosphere; (b) Influence of pressure amplitude on the maximum pressure near the hotspot under Ar atmosphere; (c) Peak temperature for 1.0 p_α and 2.0 p_α under Ar atmosphere; (d) Corresponding pressure distribution for 1.0 p_α and 2.0 p_α under Ar atmosphere.

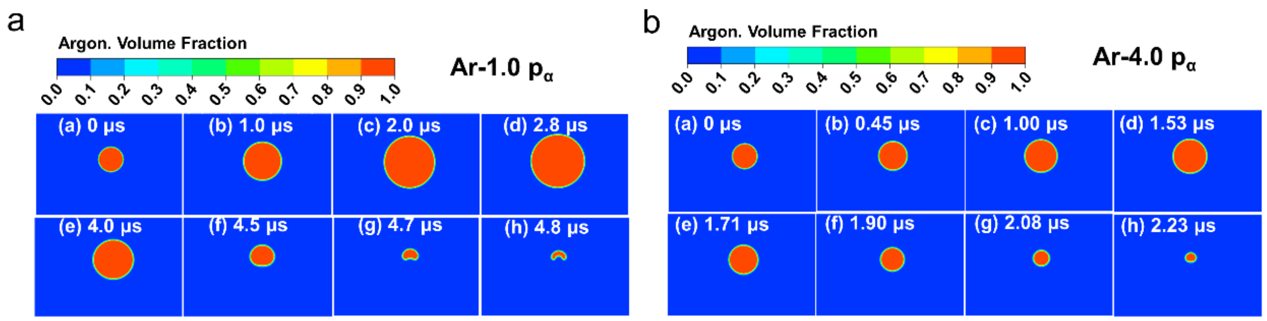

Figure S19. (a,b) Vapor volume fraction for an argon bubble under an ultrasonic condition (ultrasound frequency f=40 kHz, bubble radius R=10μm): P=1.0 p_α, P=4.0 p_α.

Under the condition of P=1.0 p_α, the Ar bubble first expands to its largest size, maintaining a spherical shape, before it begins to shrink. At the final stage, due to the effect of flow turbulence from the bottom boundary, it displays a circular ring structure. In contrast, with P=4.0 p_α, the bubble reaches its largest size earlier (at t=1.53 μs), and this bubble is notably smaller compared to that of P=1.0 p_α. The maximum temperature is recorded at t=2.24 μs, indicating that the optimal hotspot for the decomposition of water molecules happens when the bubble shrinks to the maximum extent. For both simulations, the final stage corresponds to the state point of the second rebound of the Ar bubble, respectively, indicating that the effects from the bottom boundary are diminished when the pressure amplitude is larger.

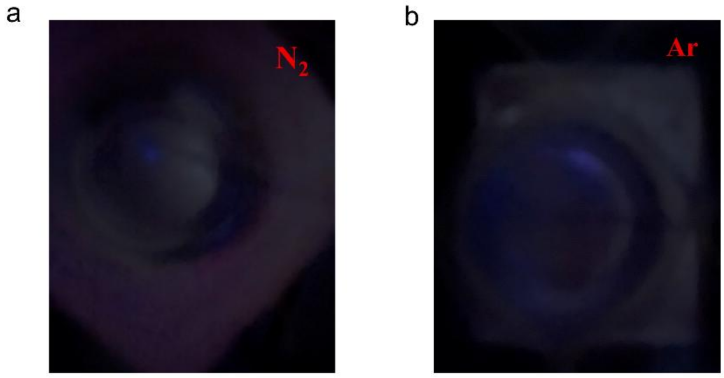

Figure S20. Photographs of SCL in different conditions. (a) N_2 and (b) Ar.

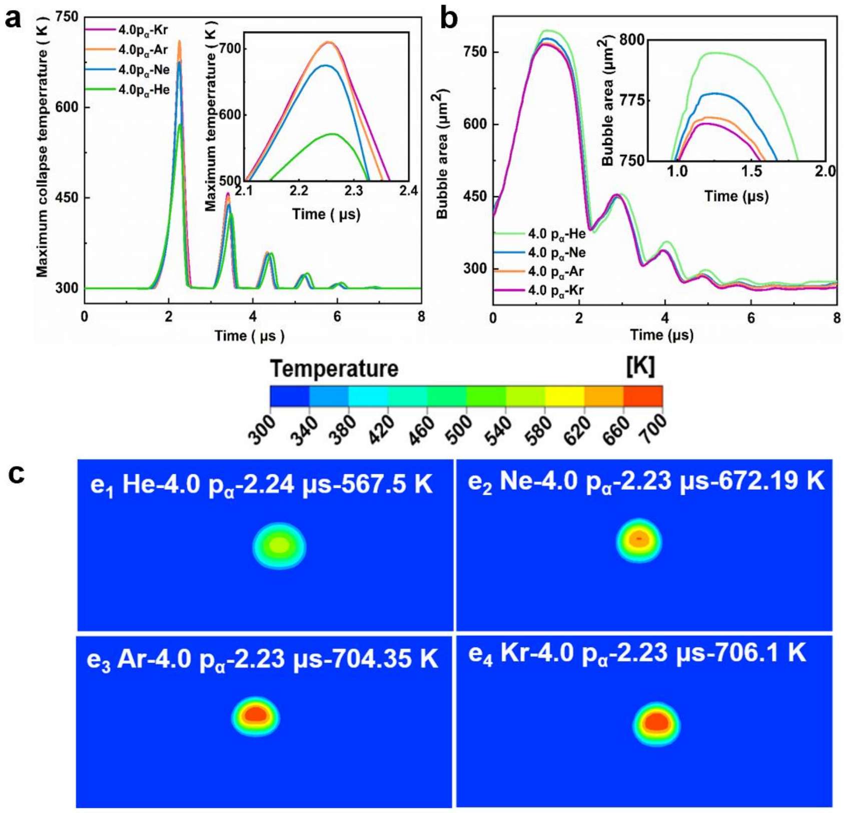

Figure S21. Physical effects of different collapsing bubbles obtained by CFD computation (ultrasound frequency f=40 kHz, bubble radius R=10 μm, pressure magnitude P=4.0 p_α) under He, Ne, Ar, and Kr atmosphere. (a) Local maximum temperature with computing time; (b) Bubble area variation with computing time; (c) Peak temperature distribution for different gases.

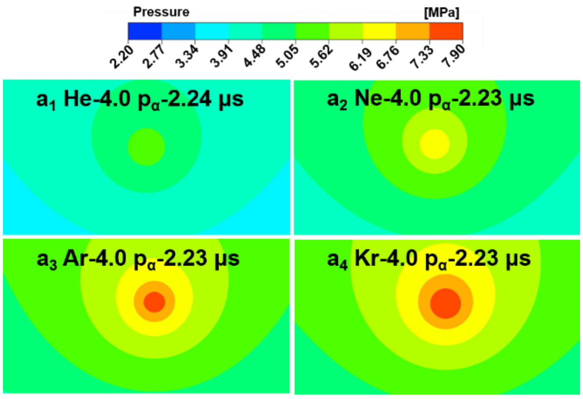

Figure S22. The corresponding local pressure under He, Ne, Ar, and Kr atmospheres.

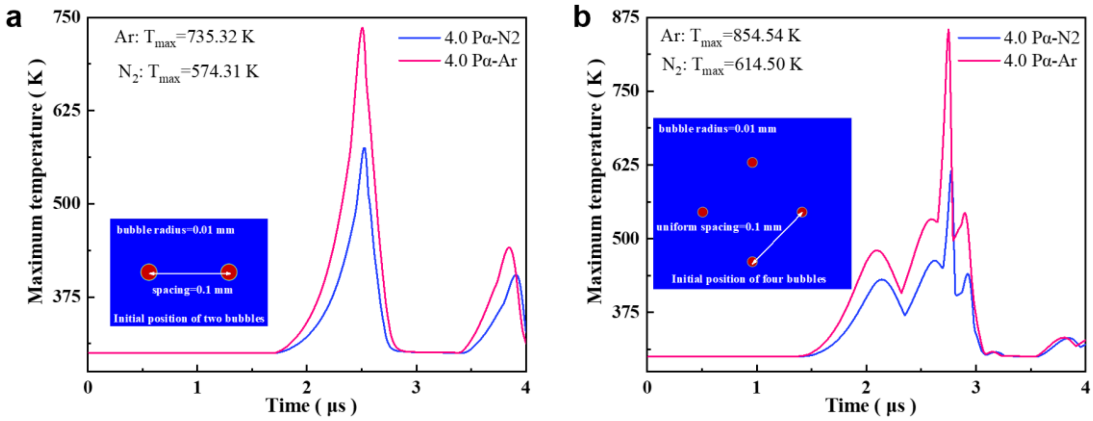

Figure S23. Multi-bubble simulation for the collapse temperature (a: two bubbles; b: four bubbles).

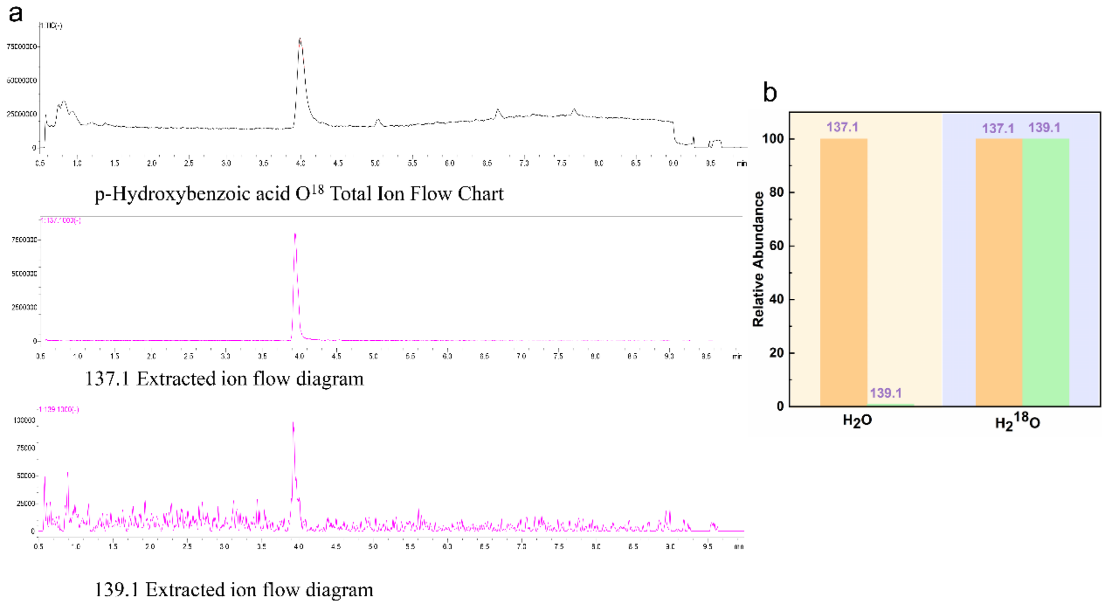

Figure S24. (a)Mass spectra of p-hydroxybenzoic acid O^{16} and O^{18}. (b). Mass spectral analysis of 4-hydroxybenzoic acid after treatment with H_2O, $H_2{}^{18}O$.

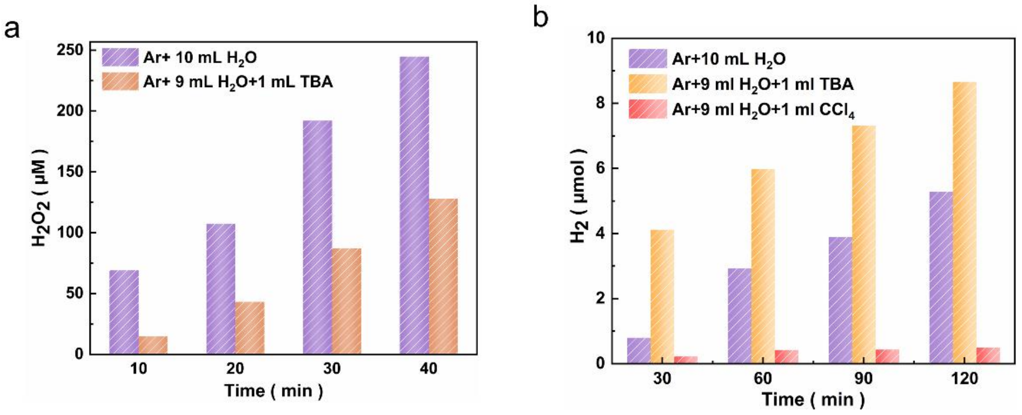

Figure S25. (a) Effect of adding tert-butanol (•OH trapping agent) on H_2O_2 generation rate. (b) Effect of addition of tert-butanol (TBA, •OH trapping agent), CCl_4 (•H) trapping agent on H_2 generation.

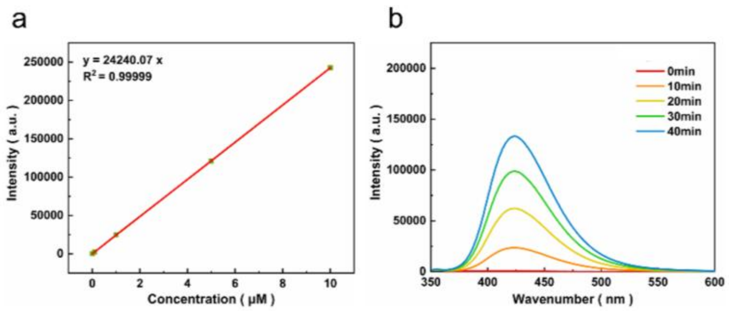

Figure S26. (a) 2-Hydroxyterephthalic acid concentration-fluorescence intensity standard curve. (b) Fluorescence spectra of 2-hydroxyterephthalic acid (HTA) at different moments under N_2 conditions.

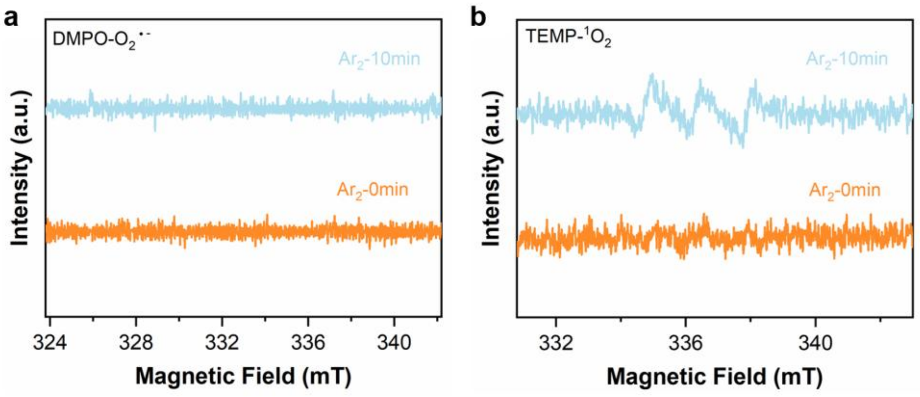

Figure S27. ESR signals of (a) $O_2^{\bullet-}$ and(b) $^1O_2$.

Section 3. Supporting Table

	Metal ion mass fraction (ppm)					W_{NPOC} (%)	Organic stabilisers (ppm)	Non-volatile residue (g/L)
	As	Fe	Ni	Cu	Pb			
H_2O_2-C	BDL	BDL	BDL	BDL	BDL	8.59×10^{-3}	/	0.09
H_2O_2-U	BDL	BDL	BDL	BDL	BDL	2.21×10^{-3}	/	0.02
Chinese National Standard (AR)	0.50	0.10	0.02	0.02	0.02	3.00×10^{-2}	/	0.60
European Pharmacopoeia (EP)	/	/	/	/	/	/	500.00	2.00
British Pharmacopoeia	/	/	/	/	/	/	250.00	2.00

Table S1. Mass fraction of metal ions and mass fraction of NPOC in H_2O_2 solution.

BDL=Below Detection Limits

Gas type	One bubble	Two bubbles	Four bubbles
Ar	710.5 K at 2.24 μs	735.32 K at 2.50 μs	854.54 K at 2.75μs
N_2	569.3 K at 2.26 μs	574. 31 K at 2.52 μs	614.50 K at 2.77μs

Table S2. CFD simulation results of internal temperature with computing time and for single bubble and multi-bubble (two bubbles and four bubbles) systems.

Section 4. References

[1] a) Q. Tian, L. Jing, S. Ye, J. Liu, R. Chen, C. H. Price, F. Fan, J. Liu, Small, 2021, 17, e2103224; b) Y. Shiraishi, T. Hagi, M. Matsumoto, S. Tanaka, S. Ichikawa, T. Hirai, Commun. Chem., 2020, 3, 169; c)Y. Shiraishi, M. Matsumoto, S. Ichikawa, S. Tanaka, T. Hirai, J. Am. Chem. Soc., 2021, 143, 12590-12599; (d) Y. Shiraishi, T. Takii, T. Hagi, S. Mori, Y. Kofuji, Y. Kitagawa, S. Tanaka, S. Ichikawa, T. Hirai, Nat. Mater., 2019, 18, 985-993.

[2] S. Zhou, D. Zhang, D. Li, H. K. Wang, C. R. Ding, J. R. Song, W. F. Huang, X. Xia, Z. W. Zhou, S. S. Han, Z. Jin, B. Yan, J. Gonzales, L. E. Via, L. Zhang, D. C. Wang, iScience, 2024, 27, 109204.

[3] J. S. Duffield, M. Lupher, V. J. Thannickal, T. A. Wynn, Annu. Rev. Pathol., 2013, 8, 241-276.

[4] N. C. Henderson, F. Rieder,T. A. Wynn, Nature, 2020, 587, 555-566.

[5] L. Song, D. Zhang, H. K. Wang, X. Xia, W. F. Huang, J. Gonzales, L. E. Via, D. C. Wang, Front. Microbiol., 2024, 14, 1301141.
